# Machine Learning Assisted Cross-Scale Hopper Design for Flowing Biomass Granular Materials

**DOI:** 10.1021/acssuschemeng.4c08938

**Published:** 2025-04-16

**Authors:** Abdallah Ikbarieh, Wencheng Jin, Yumeng Zhao, Nepu Saha, Jordan L. Klinger, Yidong Xia, Sheng Dai

**Affiliations:** † School of Civil and Environmental Engineering, Georgia Institute of Technology, 790 Atlantic Dr, Atlanta, Georgia 30332, United States; ‡ Harold Vance Department of Petroleum Engineering, Texas A&M University, 245 Spence Street, College Station, Texas 77843, United States; § Energy and Environment Science and Technology Directorate, Idaho National Laboratory, 1955 N Fermont Avenue, Idaho Falls, Idaho 83415, United States

**Keywords:** granular biomass, hopper design, flow rate, flow pattern, clogging, machine learning, biofuels

## Abstract

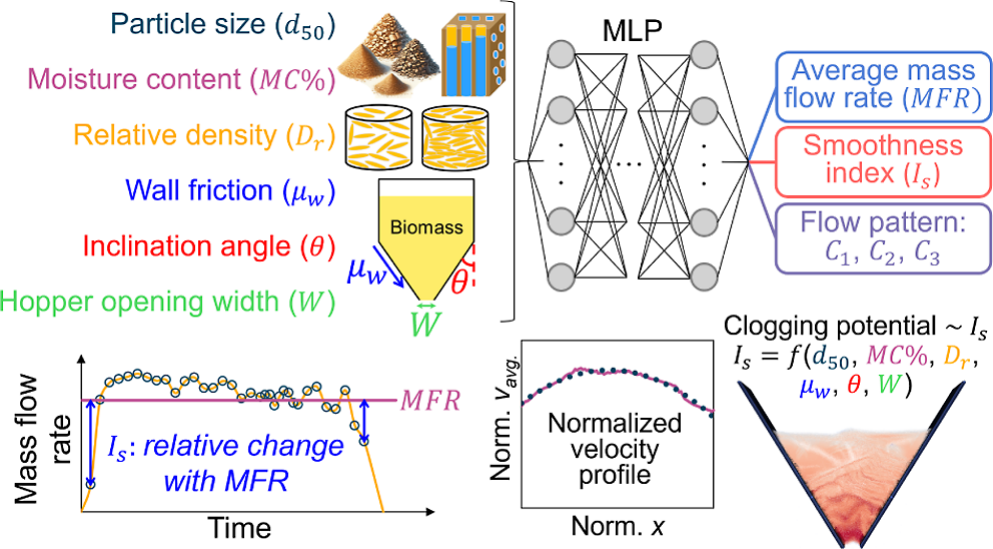

The promise of biomass-derived biofuels is often overshadowed by intricate material handling challenges such as hopper clogging and screw feeder jamming. These handling issues stem from the knowledge gap among particle-scale material properties (e.g., particle size), bulk-scale material attributes (e.g., relative density), macro-scale equipment design (e.g., hopper inclination), and flow performance (e.g., probability of clogging). This work combines physical experiments, validated numerical simulations, and data augmentation to develop a machine learning-based hopper design for flowing granular woody biomass materials. The flow behavior of granular biomass is simulated and validated against physical tests utilizing the developed smoothed particle hydrodynamics (SPH) solver and a modified hypoplastic model. A comprehensive evaluation of the flow performance, including flow rate, flow stability, and flow pattern, is conducted on an extensive data set encompassing various biomass particle sizes, moisture contents, relative densities, and hopper operating conditions. A feed-forward neural network is trained and optimized with this data set to correlate cross-scale attributes with the flow performance metrics. The results reveal promising predictive accuracy on seen and unseen data sets. Further evaluation of how various input attributes affect the predicted flow performance metrics is carried out. The results indicate that hopper opening width primarily dictates flow throughput, while relative density, wall friction, inclination angle, and hopper opening width collectively impact flow stability. Additionally, flow patterns are predominantly governed by relative density, wall friction, and inclination angle. Moreover, the clogging potential is found to be exclusively characterized by the index dedicated to flow stability. The combination of high moisture contents, dense packing, smooth wall friction, low inclination angles, and small hopper opening widths substantially elevates the risk of unstable flows and clogging. This study serves as a potent design tool for flowing milled woody biomass materials in hoppers for all stakeholders in biorefineries and equipment manufacturing.

## Abstract 2

We present a machine learning-based design tool for granular biomass flow in wedge-shaped hoppers to enhance material handling for biofuel production.

## Introduction

Biofuels present a promising alternative to conventional fossil fuels, offering the potential to significantly reduce greenhouse gas emissions.^1−11^ Biofuels are derived from biomass materials which could come from agricultural products and waste, wood, municipal solid waste, and other organic materials.^12−16^ According to the latest Billion-Ton report by the U.S. Department of Energy, more than 1 billion tons per year of biomass production is projected in the future, which could triple the current U.S. bioenergy economy, producing approximately 60 billion gallons of fuel.^17^ Despite their potential, the widespread adoption of biofuels is hindered by several challenges including preprocessing and handling of biomass feedstocks.^18,19^ Attributed to their high friction, high compressibility, irregular shapes, and low density, these feedstocks suffer from poor flowability, which is manifested as arching and jamming of biomass in handling equipment such as hoppers, feeders, and conveyors.^20−26^ Therefore, an in-depth understanding of the flow behavior of granular biomass is urgent to optimize safe handling and facilitate the design of reliable handling equipment for increased energy recovery efficiency.

In response to these operational challenges, extensive research efforts have been documented in the literature, covering both experimental and numerical studies aimed at understanding and improving the handling and processing of various biomass feedstocks and their flow behavior.^19,21,26−41^ Despite these advancements, limited studies on woody biomass handling equipment design consider the synergistic quantitative and qualitative flow characteristics, specifically flow rate, flow pattern, flow stability, and jamming risk. Consider hoppers, the most widely used handling equipment in biorefineries, their design predominantly relies on principles established in the 1960s,^42−44^ which have not been significantly updated to reflect the complexity of biomass materials. Recent studies, on the other hand, have focused on studying the impact of meso-scale properties like bulk density, compressibility, and friction angle on flow performance, primarily through experimental, numerical, or combined methods.^21,26,30,32,45^ However, these studies failed to encompass all scale attributes, including biomass particle–scale properties (e.g., particle size and moisture content), bulk-scale characteristics (e.g., relative density), and macro-scale hopper operating conditions (e.g., hopper geometry and wall friction), which collectively dictate the flow performance. In addition, these research efforts typically did not extend to predictive models or design charts specifically tailored for woody biomass materials, with the notable exception of the recent study by Lu et al.^37^ This study provided empirical guidelines for hopper design, including a formula to predict flow rate based on the particle density, particle size, critical state friction angle, hopper out-of-domain length, outlet width, inclination angle, and other material-specific coefficients. Lu et al. also introduced a design chart to differentiate mass flow from funnel flow patterns based on hopper wall friction, critical state friction angle, and hopper inclination angle. Such methodologies, while valuable, require extensive physical testing and parameter calibration. Additionally, both the mass flow rate correlation and the flow pattern chart proposed by Lu et al. omit critical factors like moisture content and relative density. Increased operational challenges stemming from these knowledge gaps and limitations highlight the need to develop comprehensive flow performance models that can ultimately achieve trouble-free hopper flow with desired flow throughput and flow pattern while minimizing the risk of clogging.

Numerical modeling tools have proven their capability to overcome the limitations of experimental testing for understanding the flow behavior of biomass materials. Physical tests, while valuable, are limited to measuring only the global flow response and fail to capture localized phenomena. Additionally, stress–strain responses of materials inside the hopper are impossible to track, hindering a thorough understanding of the material behavior. Besides these limitations, industrial-scale tests are economically infeasible for extensive testing plans.^39^ Numerical modeling, when validated against physical tests, can overcome these limitations by providing detailed, localized insights into flow dynamics and particle interactions, all while being more cost-effective and scalable for large-scale applications.^46^ Numerical tools like discrete element modeling (DEM),^47−50^ a particle-based method, is capable of modeling the complex morphology of biomass particles, while tracking their interaction individually. Nonetheless, DEM suffers from complex parametrization and high computational costs.^46,51^ Conversely, continuum-based methods, such as finite element method (FEM),^26^ finite difference method (FDM),^52^ finite volume method (FVM),^53^ material point method (MPM),^54^ and smoothed particle hydrodynamics (SPH)^55,56^ are competent at capturing the bulk mechanical behavior of biomass through robust constitutive laws. Compared to other continuum-based methods, SPH stands out as a Lagrangian mesh-free calculation method, which excels in simulating flow problems involving large deformations because it eliminates mesh distortion issues. Moreover, the application of SPH can be enhanced by leveraging GPU acceleration, which significantly boosts simulation speeds and efficiency. For instance, our recent study employed SPH with the Gudehus–Bauer (G–B) hypoplastic model^57^ to simulate woody biomass granular flow in hoppers and augers. The developed SPH solver demonstrated strong agreement between experimental and numerical flow rates. Furthermore, both shear band evolution and clogging were captured, which offered profound insights into flow patterns and the impact of physical parameters on flow performance. Building upon these advancements, integrating machine learning with existing numerical tools could unlock even greater potential, enhancing our ability to model and visualize the complete spectrum of flow phenomena in real-world applications.

Machine learning, with its ability to analyze large data sets and uncover complex patterns, offers unprecedented opportunities for enhancing predictive models and optimizing design methodologies in engineering applications.^58−61^ Specifically, in the field of granular materials, machine learning facilitates data-driven constitutive modeling that captures mechanical responses, such as stress, strain, and deformation, of materials when subjected to external loads.^62−65^ Additionally, machine learning-based models have been applied to model granular flows of idealized particles,^66−68^ showcasing their ability to learn complex relationships between variables without relying on traditional assumptions.^69^ When applied on data sets built from numerical simulations,^70,71^ machine learning-based models predict outcomes based on historical data, eliminating the need for additional parameters calibration and further computational costs.^72^ This benefit promotes model generalization on new data sets and varied material types and species.^73^ Yet, the potential applicability of machine learning techniques in understanding the flow behavior of biomass remains largely unexplored.

In this work, we combine physical experiments, validated numerical simulations, and data augmentation to establish a machine learning-based design tool for flowing granular biomass materials through wedge-shaped hoppers. This model aims to bridge the gap between biomass particle–scale properties, bulk–scale properties, macro-scale hopper design, and flow performance for trouble-free material handling. Experimental characterization data of nine biomass materials, augmented with numerical simulations, were used to calibrate the constitutive parameters of a modified version of the G–B hypoplastic model capable of capturing the flow behavior of granular biomass materials. Subsequently, hopper flow tests were simulated and validated against experimental data using our established SPH solver. Flow performance metrics were then proposed to evaluate the flow behavior of biomass through hoppers with emphasis on mass flow rate, flow pattern, and clogging potential. We performed a series of numerical simulations representing a wide range of biomass particle sizes, moisture contents, relative densities, and hopper operating conditions to evaluate the flow performance metrics necessary for building the data set. Prior to these simulations, a rigorous calibration procedure utilizing actual and synthetic physical data generated by data-driven machine learning models^65^ was employed to calibrate the modified hypoplastic model parameters of the newly proposed cases. After constructing the data set, a feed-forward neural network model was constructed and trained to predict the flow performance metrics. The model’s effectiveness was then assessed using both seen and unseen data, followed by a discussion on the physical parameters’ influence on the flow behavior. We also carried out further investigation of the clogging potential. This study serves as a novel design guide for flowing milled woody biomass materials in hoppers for biofuel production.

## Methods

We propose a machine learning model to supplement the design and operation of hoppers for flowing biomass materials. The model connects multiple scale attributes of biomass materials, including particle–scale properties (i.e., mean particle size *d*_50_, moisture content *MC* %), bulk-scale relative density (i.e., initial packing *D*_r_), and industry-scale hopper operating conditions (i.e., hopper inclination angle *θ*, hopper wall friction *μ_w_*, and hopper opening width *W*), to ultimately predicts the flow performance of biomass through wedge-shaped hoppers with emphasis on mass flow rate, flow pattern, and clogging potential. This section details the material characterization, the modified hypoplastic model calibration, numerical modeling, flow performance evaluation metrics, as well as the machine learning model construction and training.

### Experimental Characterization and Numerical Modeling

Nine whole pine samples with different particle sizes (2, 4, and 6 mm) and wet-based moisture contents (0%, 20%, and 40%) were characterized through a series of experimental characterization, which includes index tests, axial compression, and Schulze ring shear to calibrate the modified G–B hypoplastic model parameters. For more information on the samples, including the particle size distribution and particle morphology, the reader is referred to Zhao et al.^57^

The original G–B hypoplastic model^74,75^ consists of 8 constitutive parameters: granulate hardness *h*_s_, exponent *n*, and the exponent *β* all controlling the bulk compressibility of the material; critical friction angle *ϕ_c_* and exponent *α* governing the shear behavior of the material; minimum, critical, and maximum void ratios at zero pressure *e_d0_*, *e_c0_*, and *e_i0_*, respectively, all affecting the overall mechanical behavior of the material including the compressibility and shear resistance. We calibrated all these constitutive parameters using the physical characterization data. The granulate hardness *h*_s_ and exponent *n* were both calibrated using laterally confined cyclic axial compression tests. The change of bulk density *ρ_b_* with the compression stress *σ_v_* was monitored and then converted to the void ratio evolution with the mean skeleton pressure (*e* vs *p*_s_) using the relationships *e* = *ρ*_*p*_/*ρ_b_* – 1 and *p*_s_ = *σ_v_*(1 + 2*K*_0_)/3, where *ρ_p_* is the particle density, and *K*_0_ is the at-rest lateral pressure coefficient. *ρ_p_* was measured using a commercial density analyzer (Micromeridics, GeoPyc) for dry whole pines, and was used to estimate *ρ_p_* of moist samples based on *MC* %. Also, *K*_0_ was estimated from Poisson’s ratio measurements of 0.05. *h*_s_ and *n* were then calculated based on the slope of the *e* vs *p*_s_ curve. Next, the minimum void ratio *e_min_* was obtained at different loading steps from vibrating table tests,^76^ and then was projected to *e_d0_* using *h*_s_ and *n*. Subsequently, *e_c0_* was assumed to be equivalent to the maximum void ratio *e_max_*, which was obtained from rainfall tests.^77^*e_i0_*, *ϕ_c_*, *α*, and *β* were then calibrated using a coupled experimental-numerical modeling approach. Two single-element models were built in ABAQUS FEM Software^26,78,79^ to simulate the axial compression test and Schulze ring shear test.^80^ An iterative procedure was used to simultaneously vary the parameters until the error between the numerical predictions and the experimental data was minimized. Note that the previous calibrated parameters (*h*_s_, *n*, *e_d0_*, *e_c0_*) were used in these numerical simulations. Initial guesses of *e_i0_*, *ϕ_c_*, *α*, and *β* were determined as follows. Herle et al.^81^ estimated the ratio *e_i0_*/*e_max_* to be 1.2–1.3 based on the idealized material’s fabric. *e_i0_*/*e_max_* was accordingly picked from the reported range and was then further refined through the iterative calibration procedure. The G–B hypoplastic model uses the triaxial compression friction angle for *ϕ_c_*. Thus, the initial guess of *ϕ_c_* was estimated by converting the friction angle measured in the ring shear test to a triaxial compression friction angle using the concept of the Lode angle. *α* and *β* were randomly picked from their applicable reported range,^74,75,81^ and were subsequently adjusted during the error minimization process. For a detailed description of the calibration procedure, please refer to previous publications.^26,81^

**Figure 1:**
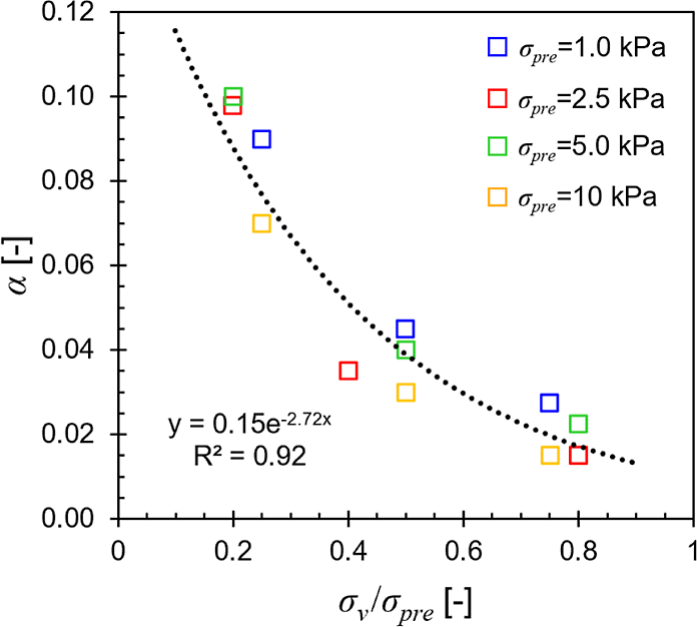
Calibrated *α* vs normalized compression stress for the 2 mm-0% sample.

**Figure 2:**
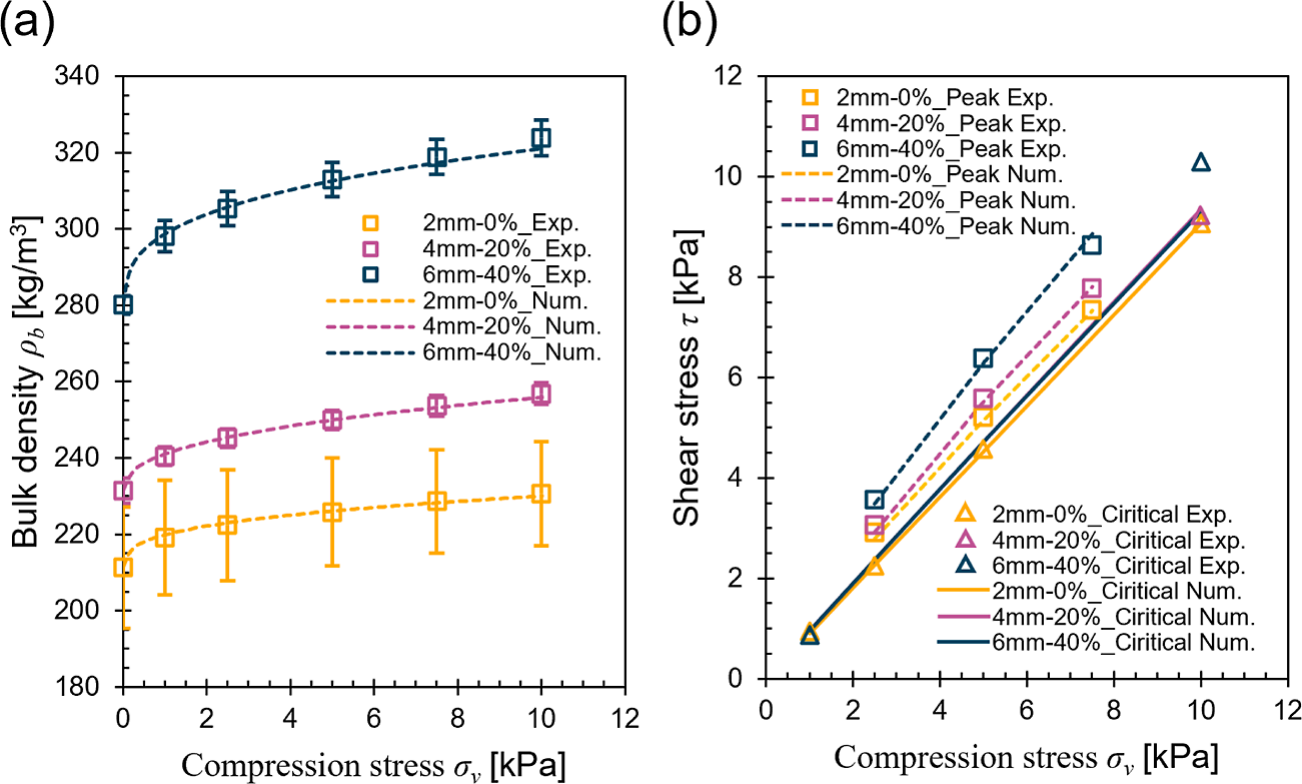
Calibration comparison of numerical prediction against the experimental measurement of the axial compression and ring shear tests for the 2 mm-0%, 4 mm-20%, and 6 mm-40% samples. (a) Bulk density *ρ_b_* vs compression stress *σ_v_*; (b) Shear stress *τ* vs compressive stress *σ_v_* for both the critical and peak states conditions.

In our analysis of raw shear stress measurements (see Supporting Information, Section S1), we observe distinct behaviors for critical and peak states of shear stress. Following the classical Mohr–Coulomb law, the shear stress *τ* measurements were fitted with the normal stress *σ_v_* measurements using a linear trend. Critical state measurements yielded a zero intercept, while peak state measurements produced a nonzero intercept. This nonzero intercept results from biomass particle interlocking. When the material is loaded to a maximum preshear compression stress *σ_pre_* in the ring shear tester, it undergoes unrecoverable plastic deformation. The material experiences shear hindrance due to the particles interlocking with subsequent shearing at lower compression stress. This behavior is manifested by a peak shear stress (i.e., strain hardening) accompanied by material dilation, followed by shear stress softening. The challenge arose when attempting to capture the interlocking effect on all tested stress levels using the original G–B hypoplastic model, which employs the exponent α in governing the peak shear stress behavior. Initial attempts using a constant *α* failed to accurately replicate the peak stress data. This discrepancy led us to refine our approach by calibrating *α* for each tested stress level, revealing a pattern where *α* decreased as the ratio *σ_v_*/*σ_pre_* increased as shown in Figure 1 exemplified using the 2 mm-0% sample. To quantitatively describe this pattern, we proposed the following exponential decay function: , where *α*_0_ is the maximum allowable value at *p*_s_ = 0, *κ* is an empirical constant defining the rate of changing *α* with *p*_s_, and *p*_0_ = 1 Pa is a reference pressure. This revised version is denoted as the modified G–B hypoplastic model in the following. After its implementation, we successfully captured the peak shear stress measurements by fine-tuning. *α_0_* and *κ* within the iterative calibration procedure discussed in the previous paragraph. Figure 2 shows a comparison between experimental data and numerical predictions of axial compression and ring shear tests demonstrated by 3 biomass samples (2 mm-0%, 4 mm-20%, 6 mm-40%) covering the whole tested particle size and moisture content ranges. Furthermore, Table 1 summarizes the calibrated parameters of the modified G–B hypoplastic model for the nine biomass samples.

**Table 1:** Calibrated Parameters of the Modified G–B Hypoplastic Model for the Whole Pine Samples

materials	*h*_s_ [MPa]	*n* [—]	*e_d0_* [—]	*e_c0_* [—]	*e_i0_* [—]	*ϕ_c_* [°]	*α_0_* [—]	*κ* × 10^–4^ [—]	*β* [—]	*ρ_p_* [kg/m^3^]
2 mm-0%	5.93	0.284	1.102	1.383	1.521	58	0.07	1.8	0.60	470.0
2 mm-20%	0.95	0.210	1.236	1.442	2.019	65	0.40	0.5	0.01	525.7
2 mm-40%	0.26	0.280	0.850	1.266	1.709	62	0.50	2.5	0.30	596.5
4 mm-0%	7.50	0.300	1.011	1.269	1.364	62	0.07	1.8	0.01	470.0
4 mm-20%	1.59	0.345	1.085	1.267	1.489	65	0.26	2.5	0.75	525.7
4 mm-40%	0.54	0.360	0.883	1.109	1.442	67	0.30	0.5	0.01	596.5
6 mm-0%	13.0	0.250	0.959	1.203	1.324	67	0.08	2.7	0.70	470.0
6 mm-20%	2.50	0.310	1.052	1.228	1.504	65	0.05	0.0	0.50	525.7
6 mm-40%	1.00	0.350	0.700	0.901	1.081	65	0.28	1.0	0.50	596.5

We observed an increase in *h*_s_ with increasing particle size. In contrast, increasing the moisture content decreased *h*_s_ (i.e., increased the bulk compressibility of the material). Additionally, *ϕ_c_* ranged from 58° to 67° for all samples. While *ϕ_c_* generally increased with increasing the particle size and moisture content, we believe that particle size and moisture content have a marginal effect on *ϕ_c_*. Indeed, the slight variations observed likely fall within the inherent variability of the tested samples. We noticed the samples’ increased compressibility with increasing moisture content enhances particles interlocking. This observation is proved with the general increase of *α_0_* with increasing the moisture content at the same particle size. Although we assumed a constant particle density among particle sizes, we noticed a discernible increase in the bulk density with increasing the particle size. This led to a reduction in the three calibrated void ratios as particle size increased. Moreover, the remaining constitutive parameters (*n*, *β*, *κ*) exhibited no clear trend with changing particle size and moisture content.

**Figure 3:**
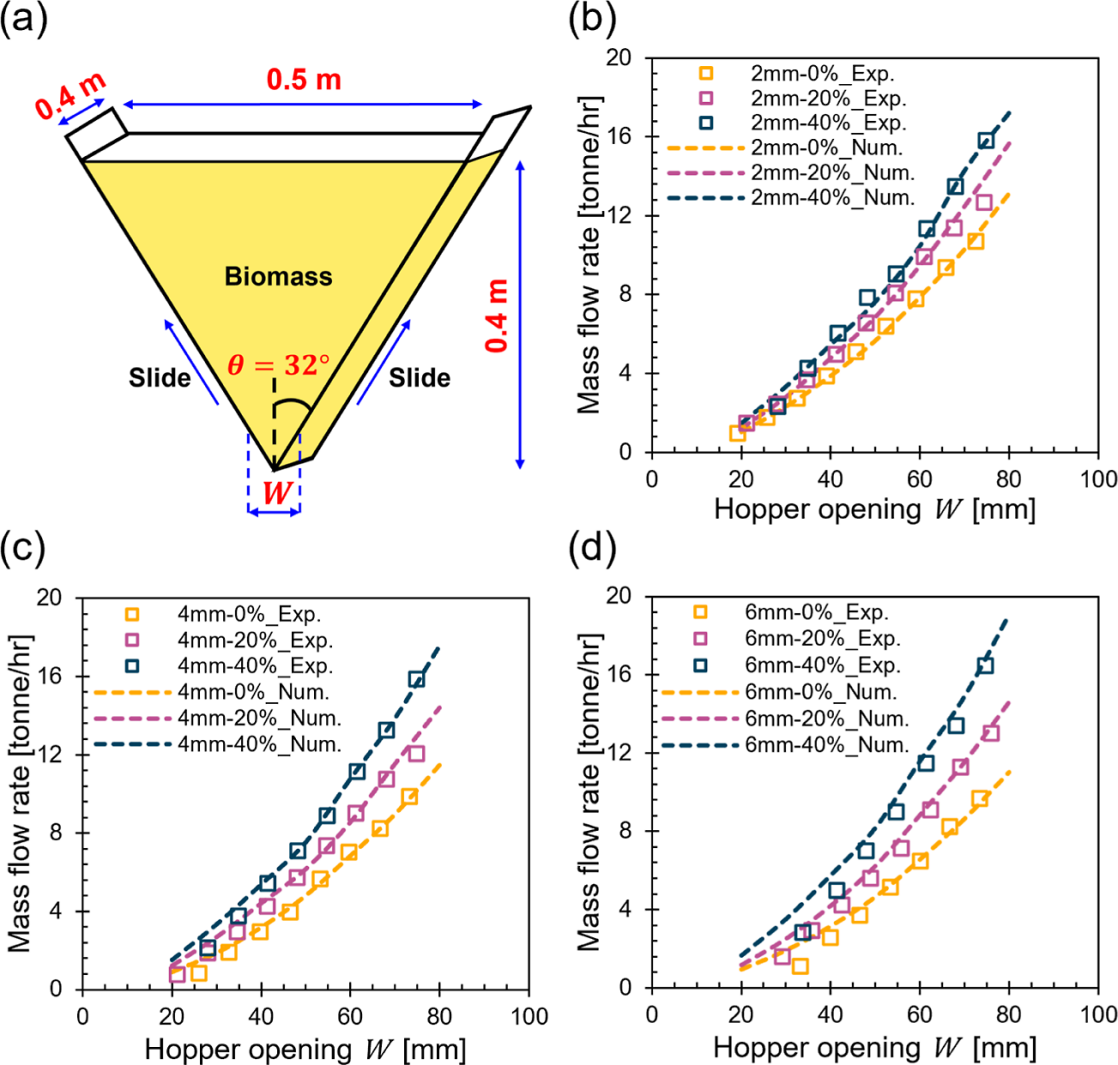
Numerical tool validation by comparing simulation results against physical measurements of hopper flow tests. (a) Sketch of the wedge-shaped hopper used for the physical experiments; Average mass flow rate from experimental measurements and numerical predictions vs hopper outlet width *W* at varying moisture content for the sample with (b) 2, (c) 4, and (d) 6 mm particle sizes.

Hopper flow tests of the nine whole pine samples were then conducted for validation against numerical modeling. Figure 3a shows the experimental setup of the wedge-shaped hopper. The hopper inclination angle *θ* is fixed as 32°, the hopper top width is 0.5 m, and the out-of-plane dimension is 0.4 m. The hopper was first charged with biomass up to a height of 0.4 m. The hopper walls were then slid up quickly to reach the desired hopper opening width *W*. Subsequently, the mass of the flowing materials was monitored with time using a high-resolution scale. For numerical modeling, we incorporated the modified G-B hypoplastic model into the established SPH solver by Zhao et al.^56^ Only a quarter of the hopper in the out-of-plane direction was simulated to reduce the computational cost, and the predicted mass flow rates were multiplied by 4 for comparison against the experimental measurements of the full-scale hopper. We adopted the recommended values of SPH numerical parameters based on the parametric study conducted by Zhao et al.^57^ An initial particle spacing *d*_p_ was set to 2 mm resulting in 1,571,040 particles for the whole simulated hopper domain. The speed of sound *c*_s_ = 80 m/s, a coefficient of artificial viscosity *α_μ_* = 0.2, a kernel size *h* = 1.25 were adopted. The coefficient of wall friction *μ_w_* was set to 0.251, corresponding to 14.1° of the wall friction angle based on experimental measurements. The material initial void ratio *e_0_* was set equal to *e_c0_* corresponding to a loose packing state. Lastly, the material was allowed to settle for 0.6 s before hopper discharge to bring the stress state to geostatic condition prior to flow. Figures 3b–d present the comparison between experimental data (scatter) and numerical predictions (smooth dashed lines) of the average mass flow rate at different hopper opening *W* for the nine whole pine samples. Results demonstrated strong agreement between the experimental measured and numerical predicted flow rates, with small discrepancies observed at smaller *W*. These discrepancies are primarily attributed to the inability of the continuum-based SPH method to fully capture the grain-scale mechanical responses.^57^ Moreover, the significant decrease in these discrepancies with increasing particle sizes further supports this justification.

### Flow Performance Evaluation

#### Average Mass Flow Rate (*MFR*)

Mass flow rate *ṁ* quantifies the amount of material expressed in mass that can be moved per unit of time. To assess the operational efficiency of hoppers over more extended periods, the average mass flow rate *MFR* is computed by aggregating the recorded mass flow rates among time steps and dividing the outcome by the number of measurements *N*. For compatibility across different hoppers with varying axial dimensions, mass flow rates have been normalized to a per unit length basis. This normalization allows MFR to be universally applicable regardless of hopper dimensions. *MFR* is expressed as follows:
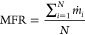
1

#### Smoothness Index (*I*_s_)

**Figure 4:**
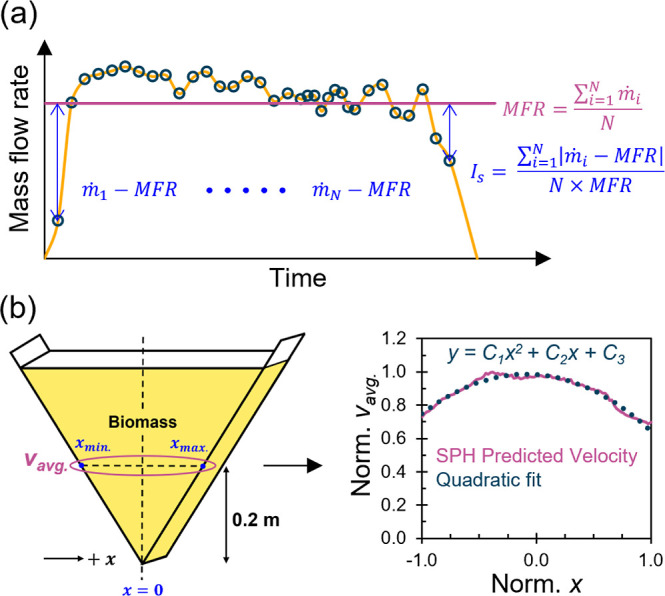
Flow performance evaluation metrics. (a) Typical mass flow rate *ṁ* time log demonstrating the average mass flow rate MFR and the smoothness index *I*_s_; (b) Typical normalized average velocity *v_avg_*_._ distribution and its corresponding quadratic fit between the normalized *x* coordinates.

Flow stability is a measure of the consistency of material delivery through handling equipment. This metric plays a crucial role in dictating equipment downtime and materials clogging potential when flow interruptions or surges are experienced. The flow stability is evaluated by monitoring the variability of the recorded mass flow rates over time. We, therefore, introduced the smoothness index *I*_s_ to quantitatively characterize the flow stability by averaging the absolute difference between ṁ and *MFR* at each time step, followed by normalizing the outcome by *MFR*. *I*_s_ is written as follows
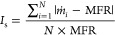
2*I*_s_ typically ranges from 0 to 1. A value approaching 0 indicates a smooth stable flow reflecting optimal operational conditions. Contrarily, a value approaching 1 is a sign of a rough/intermittent flow, suggesting an increased risk of materials clogging and potential hopper damage. Notably, *I*_s_ can exceed 1 for clogged cases, as will be discussed in a later section. Figure 4a shows a typical mass flow rate *ṁ* time series curve demonstrating *MFR* and *I*_s_ metrics.

#### Flow Pattern Polynomial Constants (*C*_1_, *C*_2_, *C*_3_)

The flow pattern is a qualitative flow property used to describe the way in which the particles flow, segregate, or compact under the influence of gravity forces. The flow pattern is dictated by the relative movement and velocity field of the particles inside the hopper. Flow patterns in hoppers are categorized mainly into mass flow pattern, in which materials at the same height move at the same speed (first-in/first-out), and funnel flow pattern, in which materials at the center move faster than the materials closer to the walls forming a stagnant zone there (first-in/last-out). Attention has been primarily paid to these two extreme flow patterns,^37,82,83^ neglecting all other possibilities. Instead, we used the SPH solver predicted velocity along the *x*-axis at mid height of the hopper to express the flow pattern (Figure 4b). We first averaged the velocity (*v_avg_*_._) of the particles among all time steps along the plane of interest. Then, we normalized the averaged velocity and the x-coordinates with their corresponding maximum values. As a result, the normalized average velocity *v_avg_*_._ and normalized x-coordinate ranged from 0.0 to 1.0 and −1.0 to 1.0, respectively. Next, we fitted the resultant normalized *v_avg_*_._ curve with a quadratic function as follows:
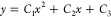
3where *C*_1_, *C*_2_, and *C*_3_ are the flow pattern quadratic constants dictating the shape of the normalized *v_avg_*_._ profile. This profile illustrates the relative velocity values across the hopper—from the walls to its centerline. The constant *C*_1_ primarily determines the opening direction and spread of the curve. As *C*_1_ decreases below zero, the spread of the curve decreases. As a result, the differences in particle velocity at the hopper center and the walls increase, aligning more with the characteristics of the funnel flow pattern. On the other hand, as *C*_1_ approaches 0, the curve flattens, and the differences between the particle’s velocity along the plane of interest decrease, suggesting a transition toward the mass flow pattern. The constant *C*_2_ dictates the horizontal location of the curve’s vertex. A *C*_2_ = 0 indicates a symmetric velocity profile about the hopper centerline, whereas a nonzero value corresponds to an asymmetric flow case. Last, *C*_3_ dictates where the curve intersects the *y*-axis.

### Data Collection and Processing

2025 simulations were proposed to construct the data set. The input variables are particle–scale properties (*d*_50_, *MC* %), relative density (*D*_r_), and hopper operating conditions (*θ*, *μ_w_*, *W*). The output variables are the flow performance evaluation metrics (*MFR*, *I*_s_, flow pattern quadratic constants: *C*_1_, *C*_2_, *C*_3_). The factorial design of the input variables comprises 5 particle sizes (*d*_50_ = 2, 3, 4, 5, 6 mm), 5 moisture contents (*MC* % = 0%, 10%, 20%, 30%, 40%), 3 relative densities (*D*_r_ = very loose, loose, dense), 3 hopper inclination angles (*θ* = 25°, 35°, 45°), 3 hopper wall friction (*μ_w_* = 0.087, 0.268, 0.577), and 3 hopper opening widths (*W* = 30, 75, 120 mm). The chosen *d*_50_ and *MC* % values reflect the typical range of particle–scale properties of processed biomass used in biofuel production. The specified relative densities represent potential materials packing encountered during the transportation and handling of materials in biorefineries. For the hopper operating conditions, the proposed *θ* and *W* values encompass the typical geometries of industrial wedge-shaped hoppers. Furthermore, the selected *μ*_*w*_ values represent smooth, textured, and rough surfaces corresponding to wall-biomass friction angles of 5°, 15°, 30°, respectively. These values cover the range of wall-biomass friction angle corresponding to different wall materials (e.g., steel, aluminum, polyethylene).^27^ For input data processing, *MC* % values were represented in fractions. In addition, relative densities were converted to numerical entries of 0, 1, and 2 for very loose, loose, and dense packing, respectively, following the ordinal encoding of categorical variables.^84,85^ Meanwhile, no further data processing was performed on the rest of the input variables.

Hopper flow simulations were conducted to establish the output variables of the proposed cases. We used the modified G–B hypoplastic constitutive law to model the mechanical behavior of the 25 proposed materials (5 *d*_50_ × 5 *MC* %). We developed a systematic approach to calibrate the constitutive law parameters for these materials. Initially, we conducted an extensive parametric study to evaluate the effect of the nine modified G–B hypoplastic model parameters on the proposed flow performance metrics. We varied each parameter within its valid range, simulated the hopper flow test, and compared the flow performance metrics of these cases against a reference case we picked. However, we disregarded *e_c0_* from the sensitivity analyses, and we used it as a reference void ratio to vary *e_d0_* and *e_i0_* as ratios (*e_d0_*/*e_c0_*, *e_i0_*/*e_c0_*).

Sensitivity analyses revealed a significant impact of *h*_s_ and *ϕ_c_* on the flow performance metrics when varied by 5 orders of magnitude and 9°, respectively. Decreasing *h*_s_ resulted in increased compressibility, thus, *MFR* decreased and *I*_s_ increased. Similarly, decreasing *ϕ_c_* induced less shear resistance, causing an increase in *MFR* and a reduction in *I*_s_. Opposite behavior was observed when *h*_s_ or *ϕ_c_* increase. Nevertheless, *h*_s_ and *ϕ_c_* had a minor effect on the shape of the fitted normalized velocity curves. Tweaking *n*, *β*, *α*_*0*_, and *κ* within their valid range for two extreme reference cases of the least and most compressible materials resulted in a marginal effect of these parameters on the flow performance metrics. In contrast, increasing the ratio *e_i0_*/*e_c0_* yielded an increase in *MFR*, while *I*_s_ varied in a complex nonlinear manner. Meanwhile, minimal changes were observed in the shape of the fitted normalized velocity curves for this case. Notably, the effect of changing *e_i0_* was less pronounced than changing *h*_s_ and *ϕ*_c_ on the flow performance metrics. Last, changing the ratio *e_d0_*/*e_c0_* produced negligible impact on the flow performance metrics. Readers are referred to the Supporting Information Section S2 for detailed results of the sensitivity analyses.

**Figure 5:**
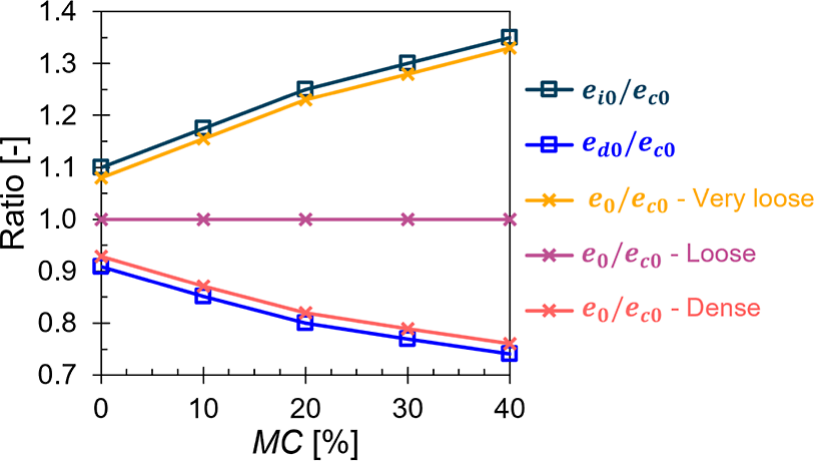
Representative normalized void ratios *e_i0_*/*e_c0_*, *e_d0_*/*e_c0_*, and *e_0_*/*e_c0_* variations with *MC* %.

Based on the findings of the sensitivity analyses and the observed overall relationships between *d*_50_, *MC* %, and the nine parameters, we categorized the parameters into 3 groups. Group 1 included *n*, *β*, *α_0_*, and *κ*, where these parameters are set to be constants equal to 0.3, 0.3, 0.1, and 1.0 × 10^–4^, respectively, for all materials. Group 2 comprised of *e_d0_*/*e_c0_* and *e_i0_*/*e_c0_* where these ratios were set to be a function of *MC* % as shown in Figure 5. It is worth mentioning that no discernible relationship was observed between *d*_50_ and *e_d0_* or *e_i0_*. Group 3 encompassed *e_c0_*, *h*_s_, and *ϕ_c_*, each calibrated differently using experimental data and synthetic data generated by data-driven machine learning models.^65^

The calibration of Groups 1 and 2 parameters involved refining the selected parameters by evaluating their combined impact on the flow performance metrics. By comparing these metrics between the adjusted cases and the nine cases that had been rigorously calibrated beforehand (Table 1), we continuously fine-tuned the parameters. This iterative process aimed to minimize the error in the flow performance metrics between the tweaked cases and the base cases. Note that *e_c0_*, *h*_s_, and *ϕ_c_* were not subject to these adjustments and retained the values established in Table 1 (see Supporting Information Section S3). In Group 3, *e_c0_* serves as a reference value for calibrating the limiting void ratios (*e_d0_*, *e_i0_*) and defining the relative density of biomass through the ratio *e_0_*/*e_c0_*. To simulate different relative densities, *e_0_*/*e_c0_* = 1.0 was used to represent loose packing. For very loose and dense packings, we simulated them by forcing *e*_0_/*e_c0_* to closely align with *e_i0_*/*e_c0_* and *e_d0_*/*e_c0_*, respectively, as demonstrated in Figure 5. To this end, we used the actual experimental data to calibrate *e_c0_*. However, for the rest of the proposed *d*_50_ and *MC* % cases lacking experimental measurements, *e_c0_* was calibrated using linear interpolation of the available data. Moreover, *h*_s_ and *ϕ_c_* were calibrated using synthetic experimental measurements generated by two distinct data-driven neural network models. These models, called the sequence and increment models, are trained on existing experimental data to predict stress–strain curves of cyclic axial compression and ring shear tests for whole pines as a function of *d*_50_ and *MC* %. In the sequential model, loading history serves as an input to Gated Recurrent Units (GRU). The GRU outputs are fed into a feed-forward neural network to predict the stress–strain of the next time-step. On the contrary, the incremental model directly inputs the current stress–strain values into a feed-forward neural network to predict stiffness components, which are used to calculate the stress–strain of the subsequent time-step. To account for material variations, both models integrate the encoded particle–scale properties into the neural networks during training. To ensure reliability, these models are validated against existing experimental data, and tested for unseen particle–scale properties and loading paths. Readers are referred to Li et al.^65^ for detailed formulation and validation of these models. Accordingly, we employed these models to generate stress–strain curves of cyclic axial compression and ring shear tests for the proposed materials. Subsequently, these curves were used to calibrate *h*_s_ and *ϕ_c_* following the detailed procedure outlined in Section 2. Note that the 7 previously calibrated parameters (*n*, *β*, *α_0_*, *κ*, *e_d0_*, *e_c0_*, *e_i0_*) were used to augment the calibration of *h*_s_ and *ϕ*_*c*_ when needed. It is also important to highlight that this established calibration procedure was applied to the 25 proposed materials to unify the approach and thoroughly validate the methodology. The finalized parameters for each of the 25 materials are detailed in Table S10 in the Supporting Information Section S4.

For numerical modeling, we used the same modeling approach and SPH numerical parameters as those outlined in Section 2. Hopper tests were simulated for a total duration of 60 s with 0.06 s time steps. Flow performance metrics were then evaluated for the simulated cases, with no additional processing applied to the data.

### Network Architecture

**Figure 6:**
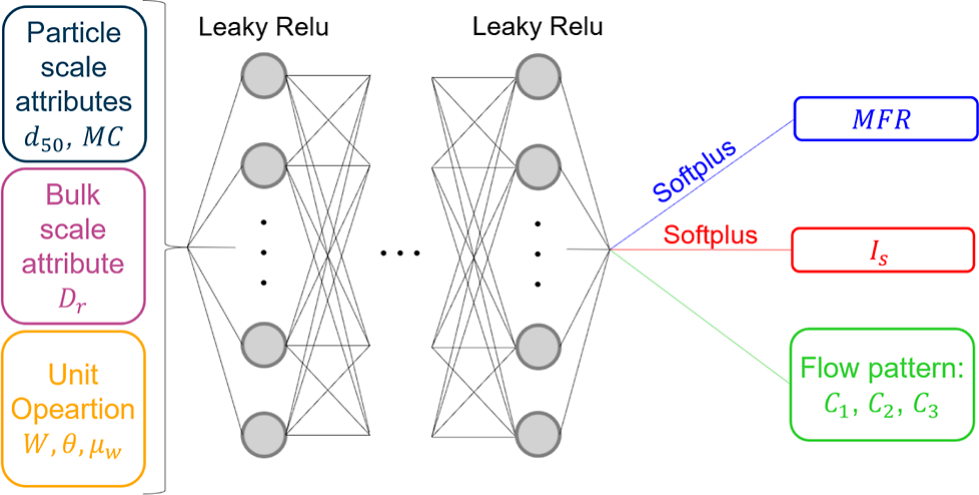
Neural network architecture used for the surrogate model.

A fully connected feed-forward neural network was established (Figure 6). The network consisted of an input layer with 6 nodes (*d*_50_, *MC* %, *D*_r_, *θ*, *μ_w_*, *W*), an output layer with 5 nodes (*MFR*, *I*_s_, *C*_1_, *C*_2_, *C*_3_), and 3 hidden layers in between. The first hidden layer contained 1000 neurons, while the second and third layers consisted of 750 and 300 neurons, respectively. The number of hidden layers and neurons was chosen to promote model generalization and prevent the risk of both overfitting and underfitting. Also, this progressive reduction of neurons in the network structure follows the geometric pyramid rule,^86,87^ which helps in simplifying the model as it moves closer to the output layer.

Each neuron takes the input from all nodes in the previous layer. Linear transformation then takes place by performing a weighted sum to its input and adding a bias term to it. These weights and biases are assigned to each fully connected layer and they are updated during training. The result is then passed through an activation function to generate the final output of the neuron, which is subsequently fed to the next layer. Indeed, activation functions play a crucial role in introducing nonlinearity in the model, which allows the network to learn complex patterns in the data. Additionally, these activation functions can help control the output range as desired. Accordingly, we first used the Relu^88^ activation function for the 3 hidden layers. However, the network suffered from the dying Relu problem.^89^ Therefore, we switched to the Leaky Relu^88^ activation function, which resolved the issue. Moreover, we applied the Softplus^90^ activation function on the first two output nodes, specifically, *MFR* and *I*_s_, to ensure that these predicted metrics consistently yield positive values, as required by their definitions.

### Training and Testing

The network was built based on PyTorch.^91^ The data set was split into 80% for training and 20% for validation. All input variables, except for *D*_r_, were standardized by centering the data around a mean of zero and scaling to a unit standard deviation. This approach demonstrated improved training performance. Also, *D*_r_ was excluded from the data standardization because it serves as a categorical variable with ordinal values of 0, 1, and 2 only. In addition, output variables were normalized using the min–max scaling technique. Thus, all data ranged from 0 to 1. This normalization is crucial for ensuring that the computed losses for each output node are on a comparable scale. Subsequently, the mean squared error (MSE) loss function was applied to the 5 output nodes. Backpropagation was then utilized to iteratively adjust the model’s parameters by computing gradients of the Average loss, which encapsulates the combined error across the five output nodes as shown in eq 4. Benefiting from the output variables normalization, the MSE loss for each individual output node ranges from 0 to 1, justifying the use of the averaging algorithm for model training. This approach helps in learning the interdependency between all input and output variables concurrently.
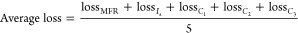
4

**Figure 7:**
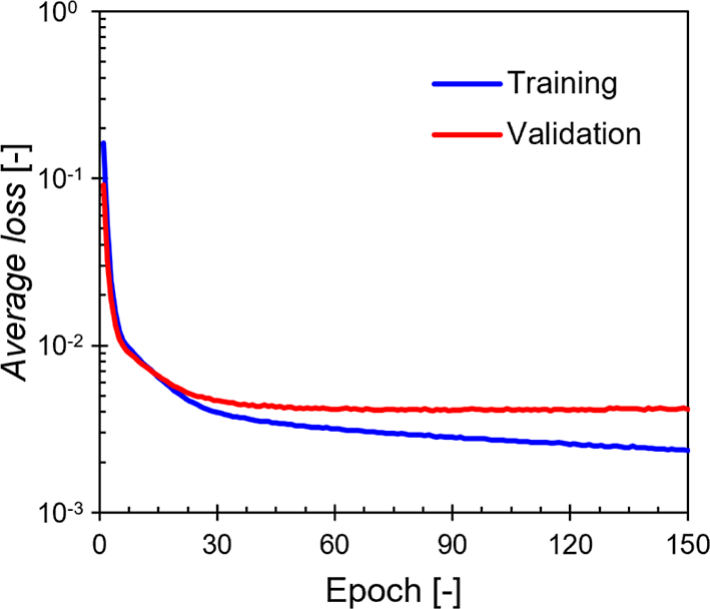
Neural network model training and validation data sets losses.

During training, we used a batch size of 30 to effectively manage the data and enhance training speed. The number of epochs was 150. Furthermore, Adam optimizer was utilized with a learning rate of 1.1 × 10^–5^. To help in preventing overfitting, L2 regularization was integrated, featuring a weight decay parameter of 5 × 10^–6^. These training parameters were optimized beforehand by evaluating the overfitting potential using cross-validation on the 80% training data. Subsequently, the optimized model was tested on the unseen 20% validation data to ensure robustness. Figure 7 illustrates the Average loss across epochs for both the training and validation data sets, with no signs of overfitting observed on the validation set.

## Results and Discussions

### Flow Performance Metrics Predictions

**Figure 8:**
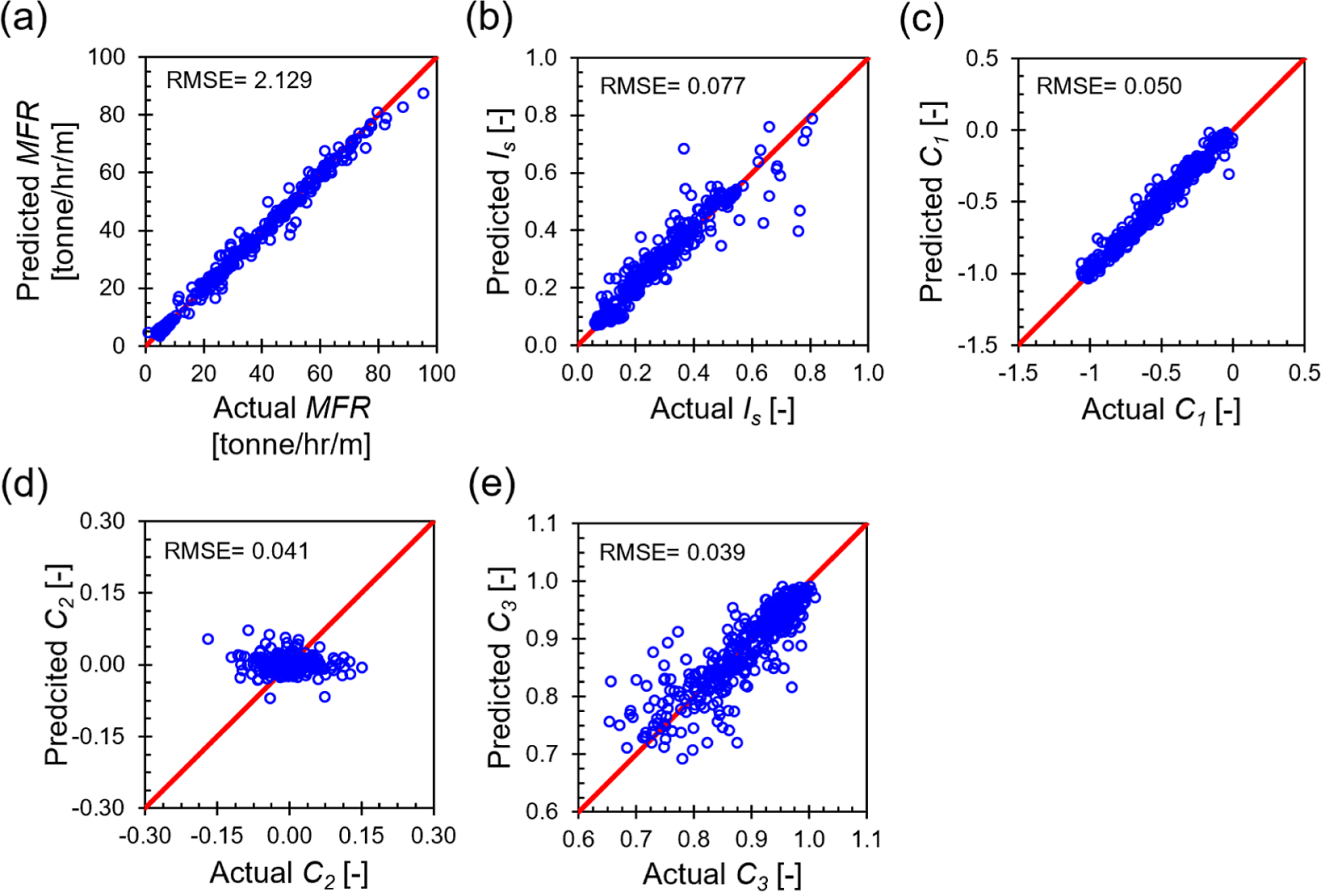
Flow performance metrics predictions for the validation set: (a) *MFR*, (b) *I*_s_, (c) *C*_1_, (d) *C*_2_, and (e) *C*_3_.

The optimized machine learning-based model was used to predict the flow performance metrics of both the training and validation sets, and the model accuracy was evaluated using the root mean square error (RMSE). Here, we present the results of the validation set as detailed in Figure 8, while the results from the training set are provided in Figure S11 in the Supporting Information Section S5 for the reader’s reference. For flow rate-specific metrics, *MFR* predictions align closely with the actual data, with a RMSE of only 2.129 tonne/h/m across the whole tested domain. Additionally, results for *I*_s_ demonstrate good predictive accuracy. However, small disparities are observed when *I*_s_ exceeds 0.6, attributable to the sparse data in this range—the data set contains only 53 instances of *I*_s_ > 0.6. This data scarcity compromises the neural network’s learning efficacy and predictive accuracy at higher *I*_s_ levels. For flow pattern quadratic constants, *C*_1_ and *C*_3_ results display good agreement between the predicted and actual data. Conversely, most *C*_2_ predictions tend to either underestimate or overestimate the actual data, though the model more accurately captures the magnitude of *C*_2_. While the RMSE for *C*_2_ is 0.041, considering only the magnitude of *C*_2_ reduces the RMSE to 0.030. This discrepancy may arise because the neural network optimizes the Averageloss across all output nodes based on the hyperparameters designed to enhance overall model performance, not specific output nodes. Importantly, accurate prediction of *C*_2_ magnitudes is sufficient to indicate an asymmetric velocity profile, regardless of the direction of asymmetry. Yet, given that all *C*_2_ values are nearly zero, users may opt to use *C*_2_ as predicted or drop it, relying solely on *C*_1_ and *C*_3_ to characterize the flow pattern.

**Figure 9:**
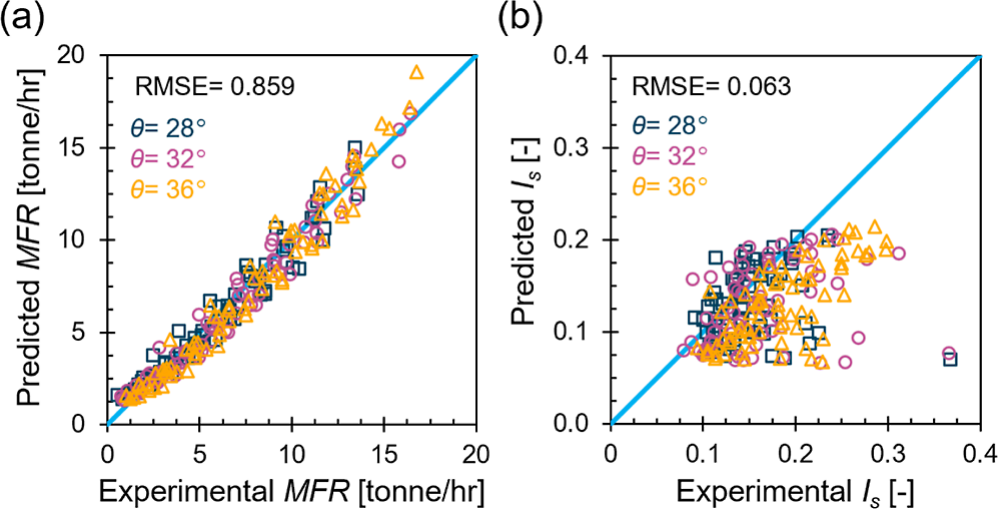
Predicted vs experimental (a) *MFR* and (b) *I*_s_ for the experimental data set.

To further validate the established framework and assess the model’s capabilities, we tested its accuracy using a separate independent data set constructed from experimental hopper flow tests consisting of 217 instances. These tests, including the data in Figure 3, were conducted on 2 mm, 4 mm, and 6 mm particle sizes; 0%, 20%, and 40% moisture contents; a hopper opening width range of 17.8–83.4 mm; and inclination angles of 28°, 32°, and 36°. Note that *μ_w_* = 0.251 and *D*_r_ = 1 (loose) were adopted for all instances. We also used an axial hopper dimension of 0.4 m to scale the machine learning predicted mass flow rates for comparison against the experimental measurements of the full-scale hopper. The experimental time series data of ṁ were analyzed to estimate *MFR* and *I*_s_. Due to our limited capacity to measure and monitor the flow pattern experimentally, we assessed the model accuracy on *MFR* and *I*_s_ only as illustrated in Figure 9. The *MFR* results indicate promising predictive accuracy with an overall RMSE of 0.859 tonne/h across tested domains. For *I*_s_, larger discrepancies between the predicted values and those estimated from experimental data have resulted. These discrepancies primarily stem from differences in the logging time steps used in the experimental and numerical *ṁ* time series data. Specifically, numerical simulations, which were used to construct the machine learning model data set, used a consistent time step of 0.06 s. Contrarily, experimental data logging was manually conducted, leading to variable time steps. Aligning time steps between experiments and numerical simulations is expected to enhance the match between the machine learning predicted and the experimentally derived *I*_s_ for this data set. Nevertheless, the relatively low RMSE of *I*_s_ corroborates the model’s capability to predict this metric effectively.

### Cross-Scale Attributes Bridging

**Figure 10:**
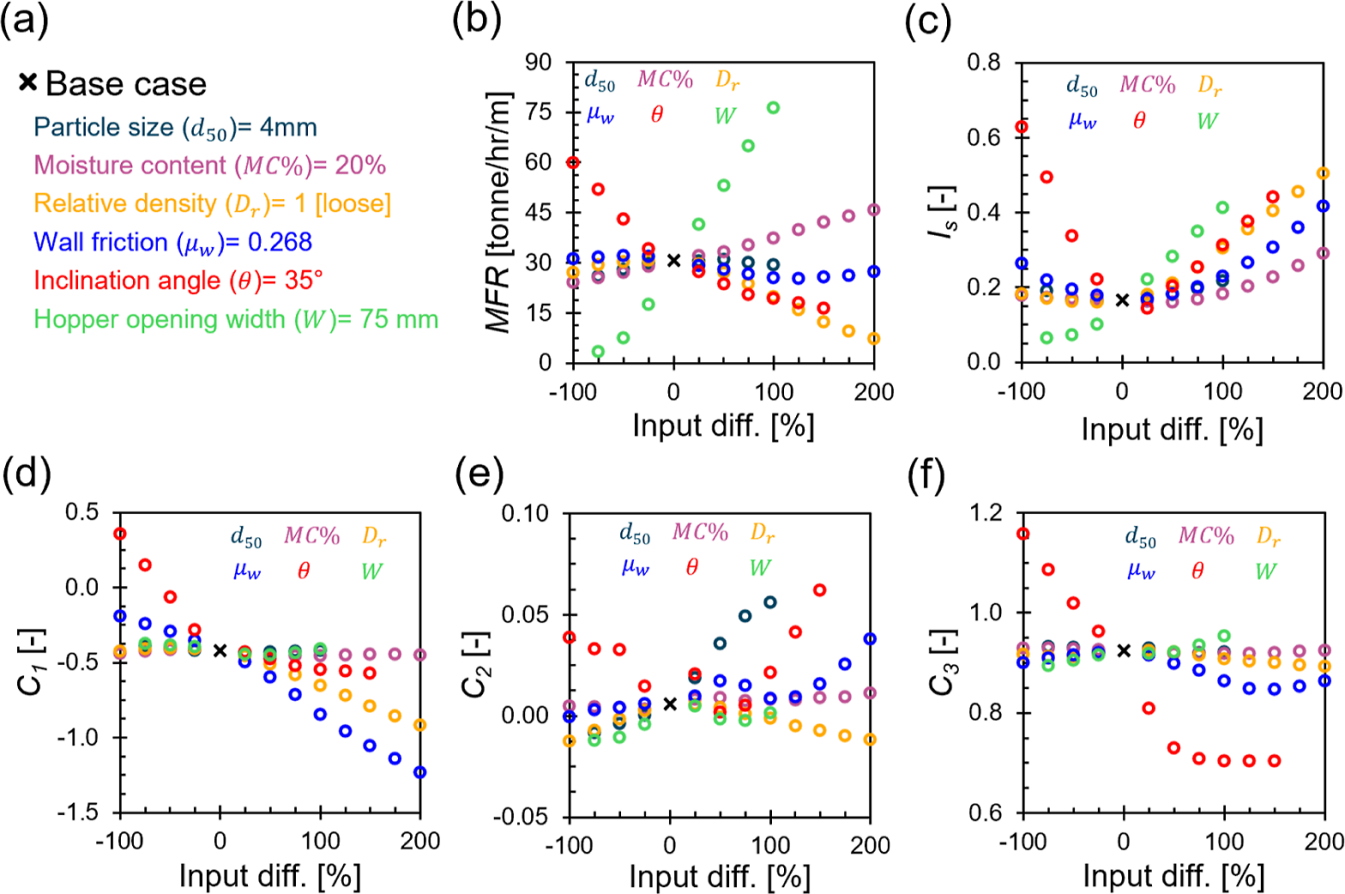
Biomass material particle–scale properties, relative density, and hopper operating conditions’ impact on flow performance metrics. (a) Base case input data; Input data change influence on (b) *MFR*, (c) *I*_s_, (d) *C*_1_, (e) *C*_2_, and (f) *C*_3_.

In this subsection, we performed a sensitivity analysis using the trained neural network model to explore how various input attributes affect the flow performance metrics. Furthermore, this sensitivity analysis aids in examining the model’s performance in the out-of-domain region. We first selected a base case as a reference, and systematically varied input parameters, one by one, within their valid physical range, as detailed in Table 2. Particularly, *d*_50_ ranged between 1 to 8 mm representing the smallest feasibly milled and largest processed biomass particles. *MC* % was adjusted between 0 and 60%, reflecting conditions from dry to extremely wet biomass. Utilizing ordinal encoding, *D*_r_ ranged from 0 to 3, spanning from very loose to very dense biomass materials. *μ_w_* ranged from 0 to 1, corresponding to a wall-biomass friction angle of 0° to 45°. *θ* was altered as 0.0° to 87.5°, covering configurations from vertical silos to high-inclination-angle hoppers. Lastly, *W* varied from 18.8 to 150.0 mm, encompassing small to large hopper opening widths. Accordingly, we predicted the flow performance metrics of these cases and tracked changes in the outputs. Figure 10a displays the base case input parameters, and Figure 10b–f summarize the results of the sensitivity analysis plotted as the predicted performance metric against the percent input difference. Note that 0% input difference denotes the base case.

**Table 2:** Base Case and Tested Range of Input Parameters Used in the Sensitivity Analyses

criteria	*d*_50_ [mm]	*MC* [%]	*D*_r_ [—]	*μ_w_* [—]	*θ* [°]	*W* [mm]
base case	4	20	1	0.268	35.0	75.0
lower bound	1	0	0	0.000	0.0	18.8
upper bound	8	60	3	1.000	87.5	150.0

Starting with *MFR* (Figure 10b), *d*_50_ shows no significant effect on the flow rate. Although an increase in particle size is expected to slightly reduce the flow rate as the effective hopper outlet area (calculated by the value of the actual size minus 2.5 times of *d*_50_) is decreased,^37^ the neural network model marginally captures this behavior. This limitation likely stems from the continuum-based approach used in numerical simulations, which were used to construct the data set for training. As mentioned earlier, the SPH solver employed cannot resolve particle–scale mechanical responses. In addition, our established base case features a hopper width of *W* = 75 mm, which is almost equivalent to about 10 times the examined particle sizes, further diminishing the expected impact of particle size on MFR. Conversely, the neural network model effectively captures the influence of moisture content on MFR, which exhibits a linear increasing trend with increasing *MC* %. For instance, increasing the *MC* % from 0 to 60% increases *MFR* by 90%, highlighting the significant effect that *MC* % has on amplifying flow rates. *D*_r_, on the other hand, demonstrates a nonlinear complex trend with *MFR*. Moving toward a very loose packing state reduces *MFR* marginally. A closer examination of the *ṁ* time series data for very loosely packed cases reveals that materials tend to flow quickly, increasing the likelihood of particle segregation. This rapid movement can lead to material collapse, which may significantly compress the materials near the outlet and subsequently reduce *MFR*. Similarly, transitioning toward a densely packed state results in a decrease in *MFR* due to increased shear resistance and particles interlocking. Notably, the reduction in *MFR* is more pronounced with denser materials compared to very loose ones, with a loose packing state being optimal for flow rate. For hopper operating conditions, *MFR* has a decreasing trend with increasing *μ_w_*. This decreasing trend is only consistent when *μ_w_* spans between 0.13 and 0.67 (∼equivalent to 8° and 38° of wall-biomass friction angle). This pattern indicates the limitation of the neural network model in capturing the expected behavior as *μ_w_* moves farther away in the out-of-domain region. Analogously, increasing *θ* from 0° to 87.5° significantly reduces *MFR* by 73%. Indisputably, *W* is the most influential parameter on *MFR*; a change in *W* from 18.8 to 150.0 mm results in a staggering increase in *MFR* by ∼2200%. Upon closer inspection, *W* and *MFR* display a bilinear directly proportional relationship, characterized by a steeper increase beyond *W* = 35 mm. These insights suggest that achieving the desired *MFR* is possible by optimizing the six input attributes, offering flexibility to adjust certain parameters when others cannot be altered due to practical constraints.

Shifting our focus to *I*_s_ (Figure 10c), we observe different trends influenced by the input attributes. *d*_50_ and *MC* % seem to have a trivial effect on *I*_s_ up to 3 mm and 40%, respectively. *I*_s_ then starts to increase beyond these values. This increase aligns with expectations: larger *d*_50_ enhances the potential for particle bridging at the hopper opening, and higher *MC* % increases material compressibility - both factors contribute to more intermittent and unstable flows. *D*_r_ pattern with *I*_s_ mirrors its correlation with *MFR*. The optimal *I*_s_ values are typically found in loosely packed materials, but *I*_s_ increases when the material packing shifts toward very loose or very dense states. In very loose conditions, faster flow rates elevate the risk of particle segregation and subsequent material collapse, while densely packed materials may form detached chunks due to stress imbalances between particles near the hopper opening and those in the upper portion of the hopper—both scenarios result in unstable flows, reflecting an increase in *I*_s_. *μ_w_* and *θ* follows a similar pattern to *D*_r_. Extremely smooth wall surfaces and low inclination angles can accelerate flow rates, leading to an increase in *I*_s_ as described before. However, as *μ_w_* and *θ* increases, *I*_s_ decreases, reaching an optimal value at 0.268 (∼equivalent to 15° of wall-biomass friction angle) and 44°, respectively. Beyond these points, *I*_s_ begins to rise again. The rise in *I*_s_ with increasing *μ_w_* is primarily attributed to increased particle segregation at the wall (i.e., near the stagnant zone) and those further away, resulting in an inconsistent, intermittent flow. In contrast, the continued increase in *I*_s_ with higher *θ* is challenging to rationalize due to sparse data in that range. It is anticipated, however, that *I*_s_ would begin to stabilize with a further increase in *θ*. Lastly, *W* exhibits a subtle increasing relationship with *I*_s_, with larger widths leading to significantly higher flow rates, thereby elevating the risk of surge flows. This increased risk is manifested with an increase in *I*_s_. Further discussion on flow stability and clogging is carried out in the Clogging Potential subsection.

For the flow pattern quadratic constant *C*_1_ (Figure 10d), particle–scale properties (i.e., *d*_50_ and *MC* %) as well as *W* show negligible effects on *C*_1_. This constant remains uniform at approximately −0.43 across all tested ranges of *d*_50_, *MC* %, and *W*, corresponding to a normalized velocity ratio of the walls over the hopper center at 0.53 (i.e., Norm *v_avg_*_._ at walls = 0.53 Norm *v_avg._* at center). This ratio indicates a flow condition intermediate between perfect mass flow and funnel flow patterns. Moreover, *D*_r_ cases spanning from very loose to loose packing states maintain the same *C*_1_ value above. However, transitioning toward denser packing decreases *C*_1_ notably up to −0.92, aligning more closely with a funnel flow pattern. This is because as relative density increases, it creates a stress imbalance where materials near the walls become densified and unable to flow, while materials near the hopper opening remain less stressed due to the lack of supporting force there. This disparity in stress distribution promotes the likelihood of a funnel flow pattern. Furthermore, *μ_w_* predominantly influences *C*_1_, exhibiting a nearly linear decreasing trend. This trend reflects increasing frictional resistance at the walls compared to the center, promoting the funnel flow pattern. For instance, as *μ_w_* rises from 0 to 1, *C*_1_ drops from −0.19 to −1.23, indicating a shift in flow pattern from ideal mass flow to ideal funnel flow. Similarly, *θ* presents a bilinear decreasing trend with *C*_1_, characterized by a steeper decrease below *θ* = 35°. As *θ* increases, the gravitational driving force component acting along the hopper walls (∼*F*_g_ cos *θ*) decreases. Concurrently, the increase in the gravitational driving force perpendicular to the hopper walls (∼*F*_g_ sin *θ*) elevates the resisting forces at the walls, calculated as *F*_g_ sin *θ* × *μ_w_*. These dynamic forces together promote funnel flow conditions, particularly as *θ* decreases.

Next, we examine *C*_2_ and *C*_3_, focusing on their responses to changes with various input parameters (Figure 10e,f). *C*_2_, which controls the flow symmetry, displays a complex nonconsistent pattern with all input parameters. Generally, the predicted *C*_2_ values range between −0.01 and 0.06, suggesting mostly asymmetric flow conditions. Nevertheless, given the trivial magnitude of *C*_2_, the resulting asymmetry in the velocity profile within the hopper is not significant, supporting the consideration of dropping *C*_2_ when characterizing the flow pattern. In cases where *C*_2_ is to be maintained, deliberate selection and adjustment of the six input parameters ensures a symmetric flow by achieving *C*_2_ = 0. The constant *C*_3_, which governs where the normalized velocity profile intersects with the *y*-axis, maintains a consistent value of approximately 0.92 across all tested variations of input parameters, excluding *θ*. Distinctively, *θ* exhibits a decreasing relationship with *C*_3_. This constant aids in interpreting the flow pattern in conjunction with *C*_1_, specifically by examining the normalized velocity ratio defined as the velocity at the wall over the center of the hopper. A simplified way to estimate this ratio is done by dropping *C*_2_ and calculating |*C*_1_ + *C*_3_|/*C*_3_, where the numerator represents the magnitude of the normalized velocity at the walls and the denominator represents that at the center. Based on this ratio, mass flow, funnel flow, and intermediate flow patterns can be distinguished: a ratio close to 0 represents an ideal funnel flow, a ratio near 1 suggests an ideal mass flow, and values between 0 and 1 indicate intermediate flow conditions. Further examination of this ratio is also presented in the next subsection.

### Clogging Potential

**Figure 11:**
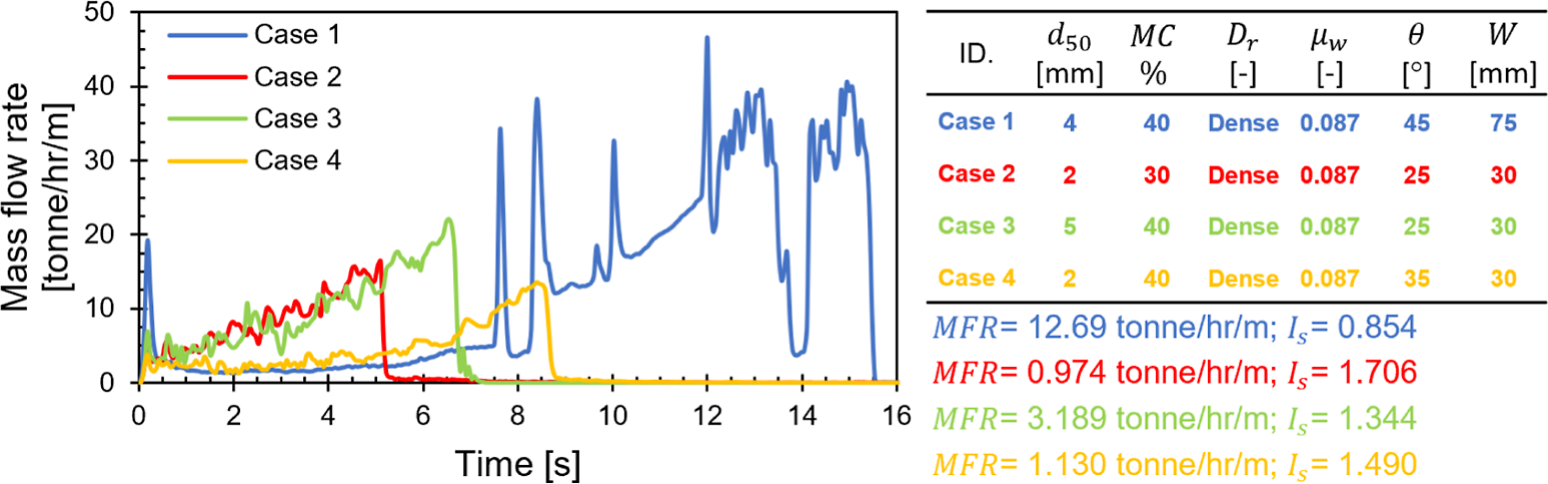
Mass flow rate time log of the intermittent unstable flow case (Case 1) and 3 clogged cases (Cases 2, 3, 4) and their corresponding *MFR* and *I*_s_ values.

This subsection delves into the factors that contribute to arching or clogging using the proposed flow performance metrics. We first attempted to incorporate the clogging potential into our neural network model by introducing a sixth output node dedicated to classifying the flow as either clogged or nonclogged. Yet, the neural network model was not able to accurately classify the flow into these two categories due to the limited number of clogged cases in the data set (only 3 clogged cases were detected). Therefore, we redirected our focus toward leveraging the flow performance metrics to better characterize the likelihood of clogging. *I*_s_ is a crucial indicator of clogging, given it has *MFR* built into its formulation, and it can assess flow stability and consistency. To this end, we analyzed the ṁ time series curves and their respective *I*_s_ values within our data set. For explanatory purposes, we present the *ṁ* time series for four distinct cases, detailing their associated attributes, *MFR*, and *I*_s_ values in Figure 11. These examples encompass one nonclogged case with a high *I*_s_ value (i.e., Case 1) and the three clogged instances identified in our data set. It is noteworthy to mention that these four cases mutually held high *MC* %, dense packing, and smooth wall friction, while differing in *d*_50_, *θ*, and *W*. In Case 1, materials initially flowed rapidly, then an arch was formed at the hopper outlet causing a slow down for nearly 7 s. Particles near the hopper outlet continued to flow slowly, causing an increase in the arch width to the point that the arch could no longer support the weight of the material above, ultimately causing it to break. Afterward, the material flowed in an intermittent manner due to several times of material collapses. In contrast, the 3 clogged cases exhibited an accelerated mass flow rate before the arch formation. The continued flow of particles near the outlet increased particles segregation due to stress imbalance. The materials at the top then collapsed, causing a complete blockage at the outlet. Unlike these clogged scenarios, the formed arch in Case 1 was unstable due to the larger outlet width *W*. In terms of *I*_s_, all four cases displayed high values, with the clogged cases exceeding a value of 1. Based on these observations, we categorize clogging potential into four levels: (i) *I*_s_ < 0.3 indicating smooth and stable flows; (ii) 0.3 < *I*_s_ < 0.6 reflecting irregular flows prone to disruptions; (iii) 0.6 < *I*_s_ < 1.0 denoting unfavorable intermittent flows; and (iv) *I*_s_ > 1.0 signifying clogged conditions. From the findings in both this and the previous subsections, it is advisable to avoid combinations of high *MC* %, dense packing, smooth *μ_w_*, and very low *θ*, particularly with smaller *W*, as the synergistic effect of these factors significantly elevates the risk of clogging. Admittedly, larger particle sizes are also likely to contribute to clogging, though this effect was not adequately captured by our neural network model, likely due to limitations inherent in the continuum-based numerical approach used in the data set construction.

We further investigated potential flow patterns that could increase the risk of clogging by examining the third and fourth categories of *I*_s_ within our data set. A detailed analysis of all instances of *I*_s_ and their respective normalized velocity ratios, |*C*_1_ + *C*_3_|/*C*_3_, reveals that these unfavorable categories cover values of this ratio spanning from 0 to 1. This observation suggests that unfavorable unstable flows and clogging can occur across various flow patterns. With the above reasoning, we conclude that *I*_s_ solely serves as an indicator for clogging.

For a practical guide on utilizing our machine learning model for selecting cross-scale attributes to achieve the desired flow performance metrics, the reader is referred to Supporting Information Section S6. This section provides detailed, step-by-step instructions for using the model to achieve satisfactory mass flow rates, flow patterns, and smooth, stable flows tailored to specific operational needs.

## Conclusions

This study presents a comprehensive examination of the flow behavior of granular biomass within wedge-shaped hoppers, providing key insights into the interplay between micro-scale material properties, bulk-scale attributes, macro-scale equipment design, and flow dynamics. Through the integration of physical tests, validated numerical models, and advanced machine learning techniques, we established a robust framework for predicting and optimizing the flow performance, including flow throughput, flow stability, and flow pattern for efficient biomass handling. The main findings of this study are as follows:

(1) Whole pine materials display significant interlocking effects during loading–unloading cycles, exacerbated by higher moisture content. This emphasizes the necessity of integrating these interlocking effects into constitutive models for a more accurate characterization of flow behavior.
(2) The machine learning-based model showcases high predictive accuracy within the training domain, with some discrepancies when input attributes move farther away in the out-of-domain region.
(3) The neural network model is not able to fully capture the effect of particle size *d*_50_ on the flow performance metrics, primarily due to limitations inherent in the continuum-based numerical approach used in the data set construction, which fails to resolve grain-scale mechanical responses adequately.
(4) Input attributes (*d*_50_, MC %, *D*_r_, μ_w_, θ, and *W*) exhibit varying positive and negative contributions on the flow performance metrics, offering opportunities to optimize flow performance according to specific operational needs.
(5) The smoothness index *I*_s_ proves to be a critical indicator of clogging potential, with a recommended value of *I*_s_ < 0.3 for smooth stable flows.
(6) Different flow patterns, including mass flow, funnel flow, and intermediate conditions, can all lead to intermittent unstable flows. This finding highlights the importance of focusing on flow-rate specific metrics to more accurately determine the likelihood of clogging.

## Data Availability

The data is available from the authors upon request.

## Supporting Information Available

Section S1; Ring shear test raw data for 2 mm-0% sample. Section S2; Modified G–B hypoplastic model parameters impact on the flow performance metrics sensitivity analysis. Section S3; Modified G–B hypoplastic model parameters grouping - impact on the flow performance metrics for parameters calibration. Section S4; Finalized modified G–B hypoplastic model parameters. Section S5; Flow performance metrics predictions. Section S6; Guide for using the machine learning model in designing cross-scale attributes (PDF)

## Nomenclature

*α*: contraction and dilation-related exponent [—]
*α_μ_*: artificial viscosity [—]
*β*, *n*: compressibility-related exponents [—]
*C*_1_, *C*_2_, *C*_3_: flow pattern quadratic constants [—]
*c_s_*: speed of sound [m/s]
*d*_50_: mean particle size [mm]
*d*_p_: initial particle spacing [mm]
*D*_r_: relative density [—]
*e_0_*: initial void ratio [—]
*e_d0_*: minimum void ratio at zero pressure [—]
*e_c0_*: critical void ratio at zero pressure [—]
*e_i0_*: maximum void ratio at zero pressure [—]
*e_min_*: minimum void ratio [—]
*e_max_*: maximum void ratio [—]
*h*: kernel size [—]
*h*_s_: granulate hardness [MPa]
*I*_s_: smoothness index [—]
*K*_0_: at-rest lateral pressure coefficient [—]
*κ*: interlocking-related exponent [—]
*ṁ*: mass flow rate [tonne/h/m]
*MC*: moisture content [%]
*MFR*: average mass flow rate [tonne/h/m]
*μ_w_*: hopper wall friction coefficient [—]
*n*, *β*: compressibility-related exponents [—]
*ϕ_c_*: critical friction angle [°]
*p*_s_: mean skeleton pressure [kPa]
*ρ_b_*: bulk density [kg/m^3^]
*ρ_p_*: particle density [kg/m^3^]
*σ_v_*: compression stress [kPa]
*σ_pre_*: preshear compression stress [kPa]
*τ*: shear stress [kPa]
*θ*: hopper inclination angle [°]
*W*: hopper outlet width [mm]
