# Supplementary material for: Machine Learning Assisted Cross-Scale Hopper Design for Flowing Biomass Granular Materials
Source: ACS Sustain Chem Eng. 2025 Apr 16;13(16):5838–51. doi: 10.1021/acssuschemeng.4c08938 (PMC12042264; doi:10.1021/acssuschemeng.4c08938)
Supplement: Supplementary file 1 — sc4c08938_si_001.pdf [file sc4c08938_si_001.pdf]

Supplementary Information

Machine learning assisted cross-scale hopper design for flowing biomass granular materials

Abdallah Ikbarieh, Wencheng Jin, Yumeng Zhao, Nepu Saha, Jordan L Klinger, Yidong Xia, and Sheng Dai

Number of pages: 20

Number of figures: 13

Number of tables: 14

S1: Ring shear test raw data for 2mm-0% sample

The shear resistance of whole pine samples is characterized using the Schulze ring shear tests performed in accordance with ASTM 6773-16 [1]. The biomass sample is poured into the ring cell using the rainfall approach, and the sample height and weight are documented. The sample is then loaded with a maximum vertical compression stress, also called the preshear compression stress, σ_{pre}. The chamber base is then rotated to shear the specimen at a constant angular velocity of 0.02 rad/min while torque is monitored, which is later converted to shear stress τ. This step continues until the shear stress reaches a steady state shear stress value (i.e., constant shear stress during rotation). Next, the rotation direction is reversed to unload the shear stress to zero. Subsequently, the sample is sheared again at a reduced compression stress σ_n to obtain the corresponding τ_n curve. Figure S1a shows the raw data of shear stress τ plotted against the shear strain γ for the 2mm-0% sample. The preshear compression stress σ_{pre} is 10 kPa, and the sample is subsequently sheared at σ_n= 2.5, 5.0, 7.5 kPa. An apparent steady state shear stress is only observed at σ_{pre} (dark blue circles), while the rest of the tested σ_n show a peak shear stress (purple circles) with no sufficient information on the critical state shear stress at these values. Therefore, we characterize the critical state shear stress from measurements obtained from several tests conducted at σ_{pre}= 1.0, 2.5, 5.0, 10.0 kPa, while the peak shear stress retains its values from σ_n= 2.5, 5.0, 7.5 kPa with a preshear compression stress σ_{pre} of 10 kPa. Those measurements are fitted using a linear trend following the classical Mohr-Coulomb law, as shown in Figure S1b. The critical state shear stress linear fit indicates a zero y-intercept, while the peak state shear stress linear fit indicates a non-zero y-intercept. This intercept is a manifestation of particles interlocking. The material undergoes unrecoverable plastic deformation when loaded and sheared at σ_{pre}. When the specimen is unloaded and sheared at σ_n, the material experiences shear frustration, reflected

as shear hardening accompanied by material dilation, followed by shear softening. We are able to incorporate this behavior into our constitutive law by introducing the changing α exponent through an exponential function defined by α_0, κ, and the mean skeleton pressure p_s, as discussed in the main manuscript.

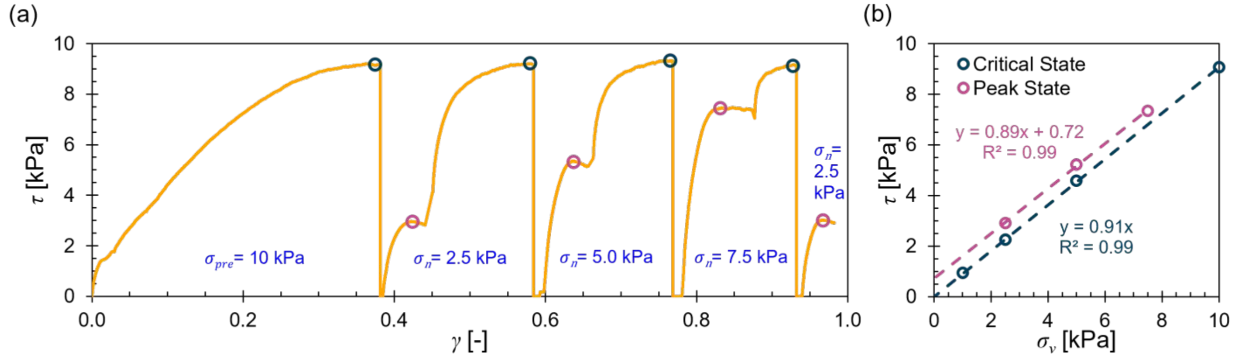

Figure S1. Ring shear test results of the 2mm-0% sample: (a) Shear stress τ vs shear strain γ at a pre-shear compression stress of σ_{pre}=10 kPa; (b) Summary of critical and peak states shear stress τ vs. the compression stress σ_v.

[1] Astm standard d6773-16, standard test method for bulk solids using Schulze ring shear tester, Tech. rep., ASTM International, West Con-shohocken, PA, 2016, www.astm.org.

S2: Modified G-B hypoplastic model parameters impact on the flow performance metrics sensitivity analysis

In the main manuscript, we discussed conducting extensive hopper flow simulations to construct the dataset used to train the neural network model. These simulations required calibrating the 9 parameters of the modified G-B hypoplastic model of all proposed cases beforehand. This sensitivity analysis served as an augmentation to the systematic approach employed to calibrate these parameters. The investigation involves varying each constitutive parameter within its valid range for whole pine samples, simulating the hopper flow test, and comparing the flow performance metrics of these cases with a reference case. Note that the impact of e_{d0} and e_{i0} are examined using the ratios e_{d0}/e_{c0} and e_{i0}/e_{c0}, respectively. All cases are simulated at a hopper opening width W of 50 mm, an inclination angle θ of 32°, and a hopper out-of-domain dimension of 0.4 m, with a total duration of 6 seconds. Here, we present the mass flow rate time log series, the corresponding flow performance metrics (MFR, I_s, C_1, C_2, C_3), and the resulting error in flow performance metrics between the tweaked and reference cases. Note that a positive error percentage indicates an increase in the metric, whereas a negative error percentage signifies a reduction. The conclusions of this sensitivity analysis are detailed in the main manuscript.

1- *Granulate hardness* h_s

The reference case is 4mm-20% having h_s= 1.6x10^3 kPa. The magnitude of h_s varies two orders of magnitude above and below the reference case. Results are summarized in Table S1 and Figure S2 below.

Table S1. Granulate hardness h_s sensitivity analysis results.

h_s [kPa]	MFR [t/hr]	Error %	I_s [-]	Error %	C_1 [-]	Error %	C_2 [-]	Error %	C_3 [-]	Error %
1.6×10^3	6.06	0.0	0.165	0.0	-0.403	0.0	-0.008	0.0	0.859	0.0
1.6×10^1	1.16	-80.9	1.340	712.1	-0.476	-18.1	-0.023	-187.5	0.582	-32.3
1.6×10^2	4.95	-18.3	0.265	60.6	-0.318	21.1	0.098	1325.0	0.869	1.2
1.6×10^4	6.54	7.9	0.126	-23.6	-0.291	27.8	0.098	1325.0	0.838	-2.4
1.6×10^5	6.76	11.6	0.133	-19.4	-0.359	10.9	-0.004	50.0	0.907	5.6

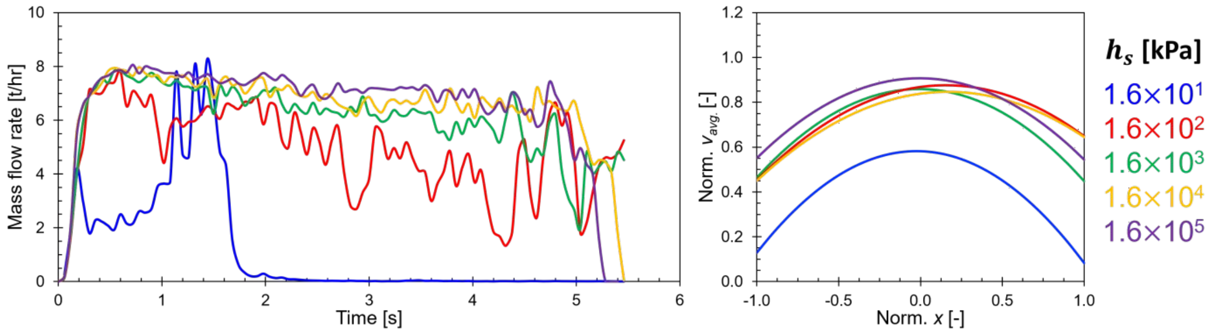

Figure S2. Mass flow rate time series and normalized velocity profile plot of granulate hardness h_s cases.

2- *Critical friction angle* ϕ_c

The reference case is 4mm-0% having ϕ_c= 62°. The reference case is compared with the lowest and highest observed ϕ_c of 58° and 67°, respectively, for whole pine samples. Results are summarized in Table S2 and Figure S3 below.

Table S2. Critical friction angle ϕ_c sensitivity analysis results.

ϕ_c [°]	MFR [t/hr]	Error %	I_s [-]	Error %	C_1 [-]	Error %	C_2 [-]	Error %	C_3 [-]	Error %
62	4.71	0.0	0.115	0.0	-0.395	0.0	-0.008	0.0	0.954	0.0

58	5.69	20.8	0.084	-27.0	-0.324	18.0	-0.015	-87.5	0.923	-3.3
67	4.13	-12.3	0.122	6.1	-0.359	9.1	-0.047	-487.5	0.973	2.0

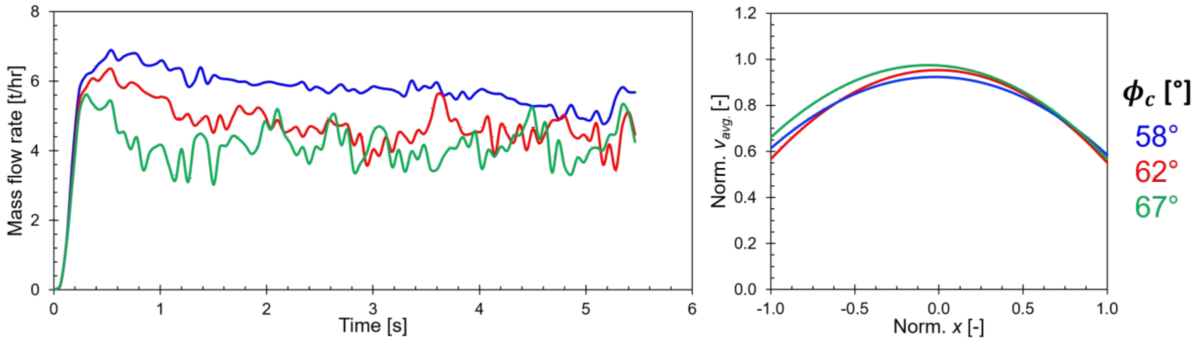

Figure S3. Mass flow rate time series and normalized velocity profile plot of critical friction angle ϕ_c cases.

3- *Exponent* n

The reference case is 2mm-0% having n= 0.284. The reference case is compared with the lowest and highest observed n of 0.210 and 0.360, respectively, for whole pine samples. Results are summarized in Table S3 and Figure S4 below.

Table S3. Exponent n sensitivity analysis results.

n [−]	MFR [t/hr]	Error %	I_s [-]	Error %	C_1 [-]	Error %	C_2 [-]	Error %	C_3 [-]	Error %
0.284	5.56	0.0	0.096	0.0	-0.357	0.0	-0.032	0.0	0.972	0.0
0.210	5.51	-0.9	0.104	8.3	-0.388	-8.7	-0.051	-59.4	0.962	-1.0
0.360	5.69	2.3	0.125	30.2	-0.293	17.9	0.059	284.4	0.957	-1.5

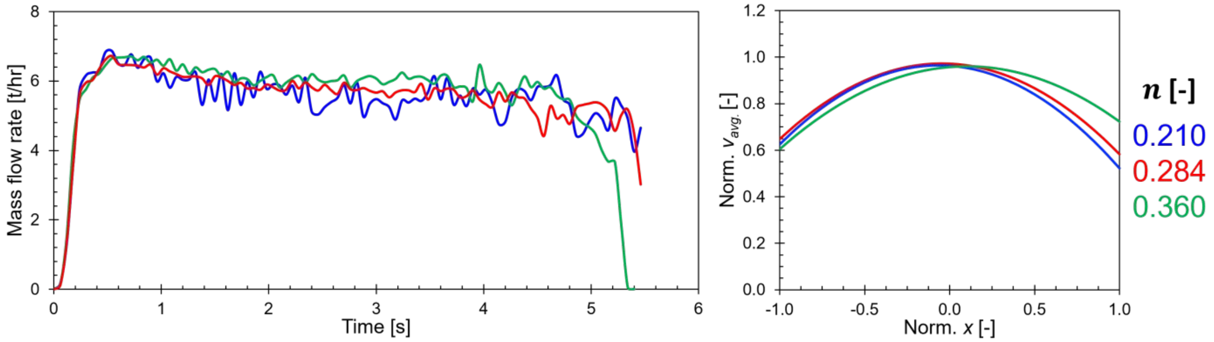

Figure S4. Mass flow rate time series and normalized velocity profile plot of exponent n cases.

4- *Exponent* β

The reference case is 2mm-0% having β= 0.6. The reference case is compared with the lowest and highest observed β of 0.01 and 0.75, respectively, for whole pine samples. Results are summarized in Table S4 and Figure S5 below.

Table S4. Exponent β sensitivity analysis results.

β [−]	MFR [t/hr]	Error %	I_s [-]	Error %	C_1 [-]	Error %	C_2 [-]	Error %	C_3 [-]	Error %
0.60	5.56	0.0	0.096	0.0	-0.357	0.0	-0.032	0.0	0.972	0.0
0.01	5.47	-1.6	0.088	-8.3	-0.339	5.0	-0.001	96.9	0.973	0.1
0.75	5.56	0.0	0.110	14.6	-0.328	8.1	-0.028	12.5	0.995	2.4

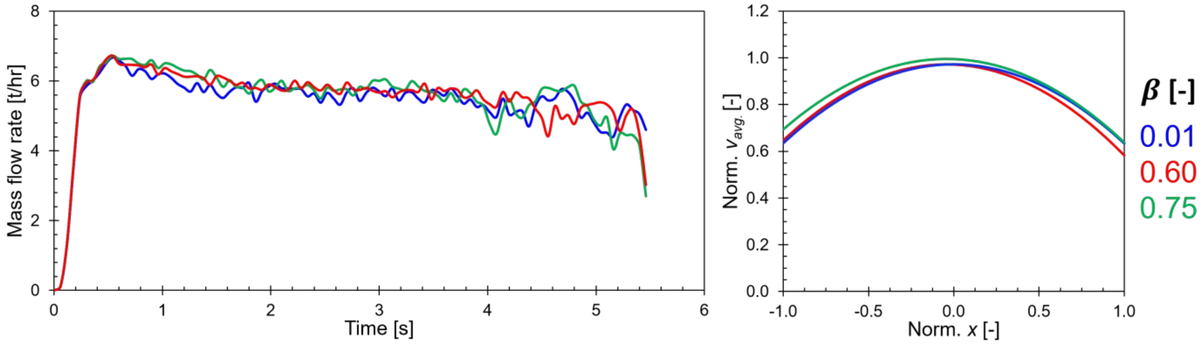

Figure S5. Mass flow rate time series and normalized velocity profile plot of exponent β cases.

5- *Constant* α_0

The reference case is 2mm-0% having α_0= 0.07. The reference case is compared with the lowest and highest observed α_0 of 0.05 and 0.50, respectively, for whole pine samples. Results are summarized in Table S5 and Figure S6 below.

Table S5. Constant α_0 sensitivity analysis results.

α_0 [−]	MFR [t/hr]	Error %	I_s [-]	Error %	C_1 [-]	Error %	C_2 [-]	Error %	C_3 [-]	Error %
0.07	5.56	0.0	0.096	0.0	-0.357	0.0	-0.032	0.0	0.972	0.0
0.05	5.56	0.0	0.091	-5.2	-0.412	-15.4	0.000	100.0	0.960	-1.2
0.50	5.56	0.0	0.142	47.9	-0.402	-12.6	0.037	215.6	0.970	-0.2

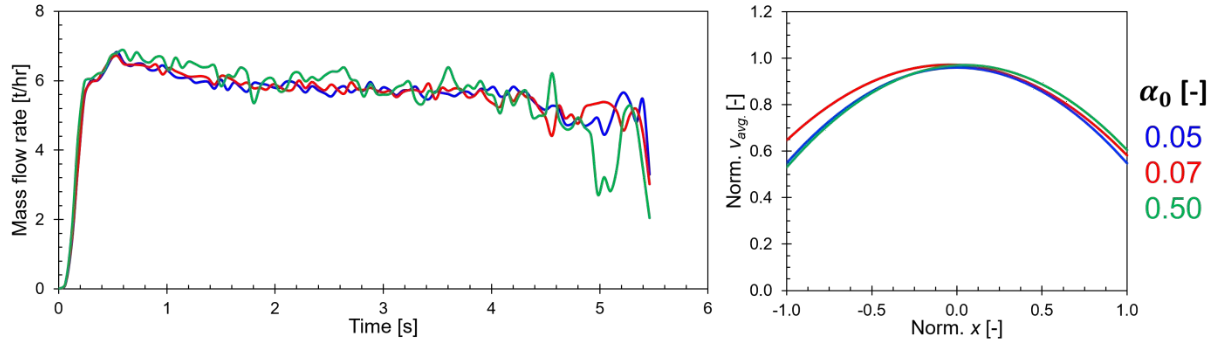

Figure S6. Mass flow rate time series and normalized velocity profile plot of constant α_0 cases.

6- *Exponent* κ

The reference case is 2mm-0% having κ= 1.8×10^{-4}. The reference case is compared with the lowest and highest observed κ of 0.0 and 2.7×10^{-4}, respectively, for whole pine samples. Results are summarized in Table S6 and Figure S7 below.

Table S6. Exponent κ sensitivity analysis results.

κ×10^{-4} [−]	MFR [t/hr]	Error %	I_s [-]	Error %	C_1 [-]	Error %	C_2 [-]	Error %	C_3 [-]	Error %
1.8	5.56	0.0	0.096	0.0	-0.357	0.0	-0.032	0.0	0.972	0.0
0.0	5.55	-0.2	0.089	-7.3	-0.399	-11.8	0.026	181.3	0.976	0.4
2.7	5.53	-0.5	0.094	-2.1	-0.345	3.4	-0.037	-15.6	0.882	-9.3

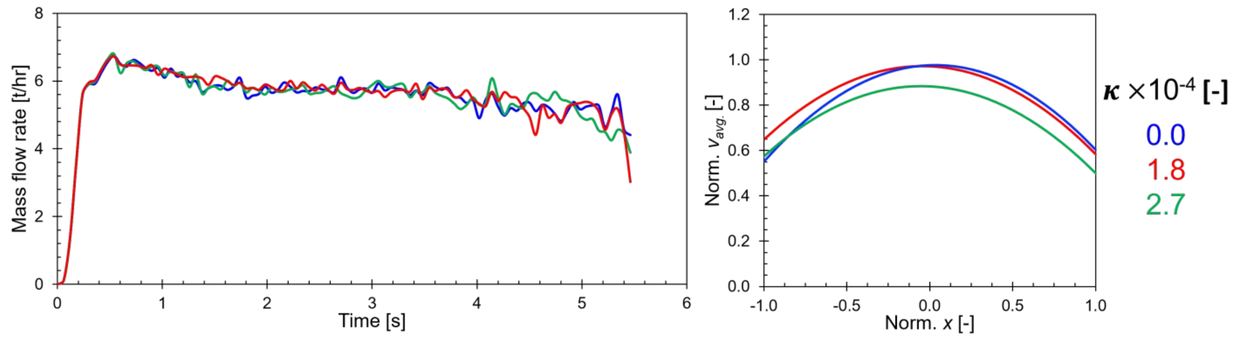

Figure S7. Mass flow rate time series and normalized velocity profile plot of exponent κ cases.

7- e_{d0}/e_{c0} *ratio*

The reference case is 2mm-0% having e_{d0}/e_{c0}= 0.797. The reference case is compared with the lowest and highest observed ratios of 0.671 and 0.855, respectively, for whole pine samples. Results are summarized in Table S7 and Figure S8 below.

Table S7. e_{d0}/e_{c0} ratio sensitivity analysis results.

e_{d0} $/e_{c0}$ $[-]$	MFR [t/hr]	Error %	I_s [-]	Error %	C_1 [-]	Error %	C_2 [-]	Error %	C_3 [-]	Error %
0.797	5.56	0.0	0.096	0.0	-0.357	0.0	-0.032	0.0	0.972	0.0
0.671	5.56	0.0	0.121	26.0	-0.349	2.2	0.001	103.1	0.958	-1.4
0.855	5.56	0.0	0.098	2.1	-0.366	-2.5	-0.011	65.6	0.965	-0.7

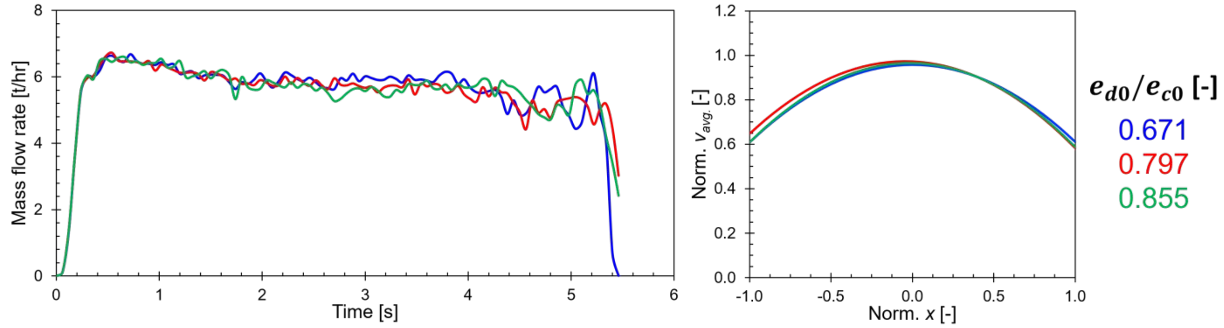

Figure S8. Mass flow rate time series and normalized velocity profile plot of e_{d0}/e_{c0} ratio cases.

8- e_{i0}/e_{c0} *ratio*

The reference case is 2mm-0% having e_{i0}/e_{c0}= 1.10. The reference case is compared with the lowest and highest observed ratios of 1.08 and 1.40, respectively, for whole pine samples. Results are summarized in Table S8 and Figure S9 below.

Table S8. e_{i0}/e_{c0} ratio sensitivity analysis results.

e_{i0} $/e_{c0}$ $[-]$	MFR [t/hr]	Error %	I_s [-]	Error %	C_1 [-]	Error %	C_2 [-]	Error %	C_3 [-]	Error %
1.10	5.56	0.0	0.096	0.0	-0.357	0.0	-0.032	0.0	0.972	0.0
1.08	5.35	-3.8	0.100	4.2	-0.314	12.0	0.076	337.5	0.961	-1.1
1.40	6.41	15.3	0.111	15.6	-0.343	3.9	0.045	240.6	0.961	-1.1

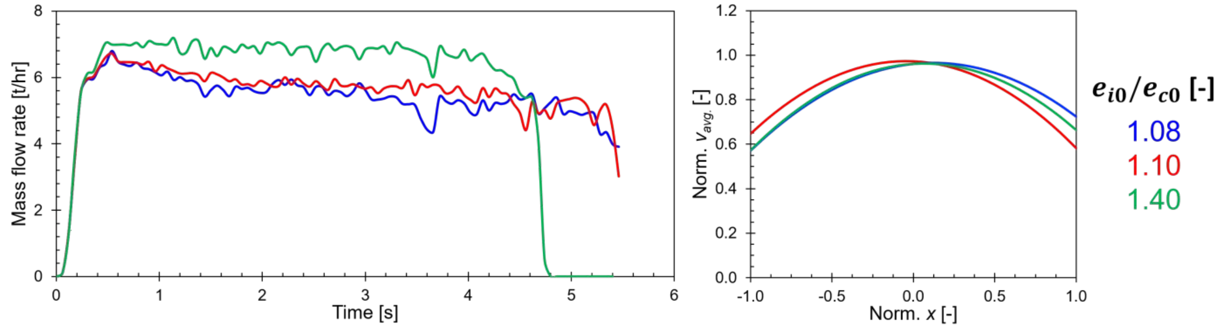

Figure S9. Mass flow rate time series and normalized velocity profile plot of e_{i0}/e_{c0} ratio cases.

S3: Modified G-B hypoplastic model parameters grouping - impact on the flow performance metrics for parameters calibration

Following the sensitivity analysis performed in section S2, we examine the impact of grouping n, β, α_0, and κ as Group 1, and the ratios e_{d0}/e_{c0} and e_{i0}/e_{c0} as Group 2, on the flow performance metrics for calibration purposes as discussed in the main manuscript. We tweaked the parameters simultaneously, evaluated the flow performance metrics of these tweaked cases, and compared them with the ones obtained from the cases established in Table 1 in the main manuscript (reference cases). These cases are simulated at a hopper opening width W of 50 mm and an inclination angle θ of 32°, with a total duration of 6 seconds. The parameters in each group are adjusted until the error between the flow performance metrics of the tweaked cases and the reference cases is minimized. Here, we present the comparison results between the reference cases and the finalized tweaked cases in Table S9. Moreover, the mass flow rate time log series and normalized velocity profile plots are illustrated in Figure S10. These cases feature n= 0.3, β= 0.3, α_0= 0.1, and κ= 1.0×10^{-4}, while e_{d0}/e_{c0} and e_{i0}/e_{c0} are varied as a function of $MC\%$ as shown in Figure 5 in the main manuscript. Note that h_s, ϕ_c, and e_{c0} of the tweaked cases retained their values from the reference cases.

The optimized constitutive parameters of Groups 1 and 2 feature a maximum absolute error percentage of 11.9%, 44.8%, 25.8%, 292.0%, and 21.5% for MFR, I_s, C_1, C_2, and C_3, respectively. Higher error percentages for I_s and C_2 are deemed acceptable, as these metrics inherently represent small magnitudes where minor variations can lead to proportionally larger percentage errors. Additionally, all I_s values are below 0.3, indicative of smooth, stable flows. Similarly, C_2 values are consistently near zero, highlighting their negligible impact on the normalized velocity profile. Therefore, the acceptance of larger error percentages for these metrics is justified, given their minor role in the overall analysis at this stage.

Table S9. Flow performance metrics comparison between the finalized tweaked cases and the reference cases.

Material	Case	MFR [t/hr]	Error %	I_s [-]	Error %	C_1 [-]	Error %	C_2 [-]	Error %	C_3 [-]	Error %
2mm-0%	Ref.*	5.56	0.0	0.096	0.0	-0.357	0.0	-0.032	0.0	0.972	0.0
	Twk.‡	5.62	1.1	0.112	16.7	-0.265	25.8	0.036	212.5	0.993	2.2
2mm-20%	Ref.	6.58	0.0	0.186	0.0	-0.374	0.0	0.044	0.0	0.826	0.0
	Twk.	5.8	-11.9	0.119	-36.0	-0.299	20.1	0.021	-52.3	1.004	21.5
2mm-40%	Ref.	7.42	0.0	0.232	0.0	-0.370	0.0	-0.086	0.0	0.893	0.0
	Twk.	7.59	2.3	0.128	-44.8	-0.408	-10.3	-0.036	58.1	0.879	-1.6
4mm-0%	Ref.	4.71	0.0	0.115	0.0	-0.395	0.0	-0.008	0.0	0.954	0.0
	Twk.	5.27	11.9	0.120	4.3	-0.383	3.0	-0.020	-150.0	0.981	2.8
4mm-20%	Ref.	6.05	0.0	0.165	0.0	-0.403	0.0	-0.008	0.0	0.859	0.0
	Twk.	6.43	6.3	0.099	-40.0	-0.342	15.1	0.001	112.5	0.854	-0.6
4mm-40%	Ref.	7.38	0.0	0.172	0.0	-0.331	0.0	0.050	0.0	0.883	0.0
	Twk.	7.69	4.2	0.129	-25.0	-0.323	2.4	-0.096	-292.0	0.954	8.0
6mm-0%	Ref.	4.54	0.0	0.126	0.0	-0.418	0.0	0.054	0.0	0.938	0.0
	Twk.	4.75	4.6	0.136	7.9	-0.395	5.5	-0.011	-120.4	0.971	3.5
6mm-20%	Ref.	6.12	0.0	0.093	0.0	-0.429	0.0	-0.044	0.0	0.968	0.0
	Twk.	6.6	7.8	0.093	0.0	-0.440	-2.6	-0.003	93.2	0.974	0.6
6mm-40%	Ref.	8.03	0.0	0.141	0.0	-0.398	0.0	0.053	0.0	0.948	0.0
	Twk.	8.83	10.0	0.110	-22.0	-0.374	6.0	0.014	-73.6	0.917	-3.3

*Ref.: Reference case
‡Twk.: Tweaked case

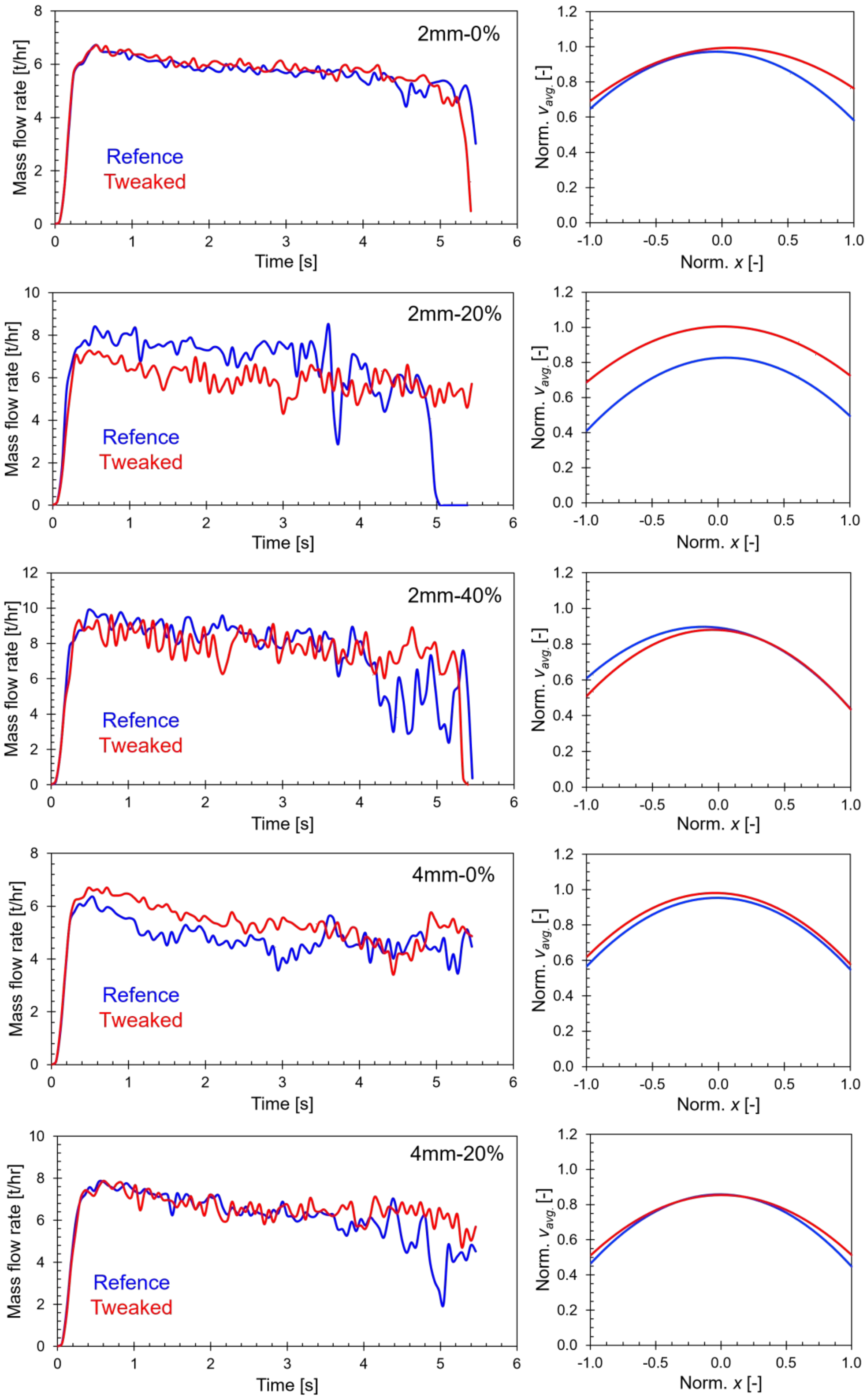
2mm-0%
2mm-20%
2mm-40%
4mm-0%
4mm-20%
Refence
Tweaked
Mass flow rate [t/hr]
Time [s]
Norm. $v_{avg.}$ [-]
Norm. x [-]

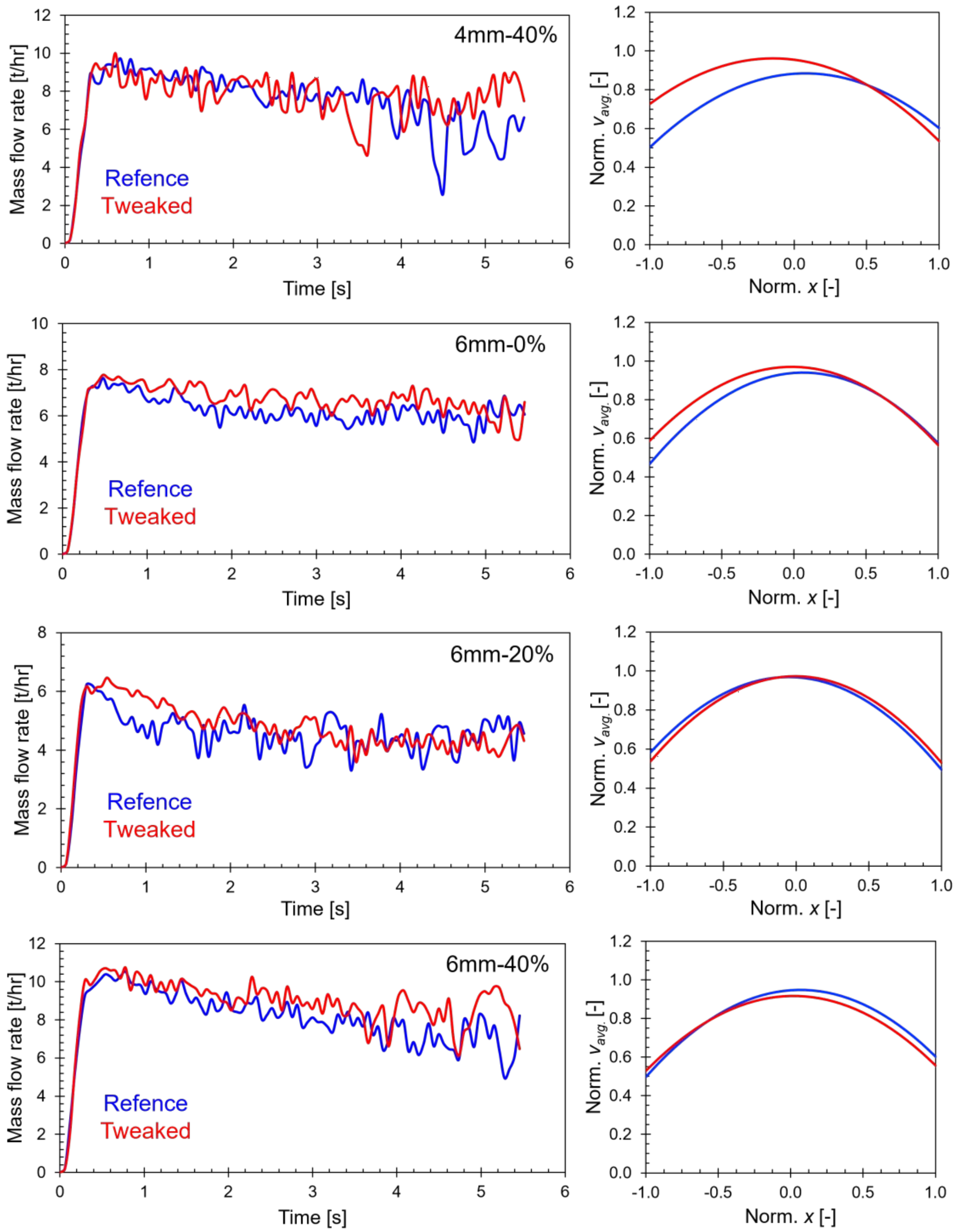

Figure S10. Mass flow rate time series and normalized velocity profile plots of the reference and tweaked cases for the nine whole pine samples.

S4: Finalized modified G-B hypoplastic model parameters

Table S10. The finalized parameters of the modified G-B hypoplastic model for the 25 materials used in the machine learning-based model dataset construction.

d_{50} [mm]	$MC\%$	h_s [MPa]	ϕ_c [°]	e_{c0} [-]	n [-]	β [-]	α_0 [-]	$\kappa\times10^{-4}$ [-]	e_{d0} [-]	e_{i0} [-]	ρ_p [kg/m^3]
2	0	6.05	58.0	1.383	0.3	0.3	0.1	1.0	1.257	1.521	470
2	10	3.27	61.5	1.406	0.3	0.3	0.1	1.0	1.197	1.652	496.3
2	20	0.91	65.0	1.442	0.3	0.3	0.1	1.0	1.154	1.803	525.7
2	30	0.61	63.5	1.336	0.3	0.3	0.1	1.0	1.028	1.737	558.9
2	40	0.26	62.0	1.266	0.3	0.3	0.1	1.0	0.938	1.709	596.4
3	0	6.32	60.0	1.325	0.3	0.3	0.1	1.0	1.205	1.458	470
3	10	3.94	62.5	1.331	0.3	0.3	0.1	1.0	1.133	1.564	496.3
3	20	1.37	65.0	1.351	0.3	0.3	0.1	1.0	1.081	1.689	525.7
3	30	0.79	64.8	1.251	0.3	0.3	0.1	1.0	0.962	1.626	558.9
3	40	0.41	64.5	1.185	0.3	0.3	0.1	1.0	0.878	1.600	596.4
4	0	7.96	62.0	1.269	0.3	0.3	0.1	1.0	1.154	1.396	470
4	10	4.18	63.5	1.261	0.3	0.3	0.1	1.0	1.073	1.482	496.3
4	20	1.56	65.0	1.267	0.3	0.3	0.1	1.0	1.014	1.584	525.7
4	30	1.1	66.0	1.172	0.3	0.3	0.1	1.0	0.902	1.524	558.9
4	40	0.49	67.0	1.109	0.3	0.3	0.1	1.0	0.821	1.497	596.4
5	0	9.74	64.5	1.236	0.3	0.3	0.1	1.0	1.124	1.360	470
5	10	5.8	64.8	1.235	0.3	0.3	0.1	1.0	1.051	1.451	496.3
5	20	2.04	65.0	1.247	0.3	0.3	0.1	1.0	0.998	1.559	525.7
5	30	1.33	65.5	1.100	0.3	0.3	0.1	1.0	0.846	1.430	558.9
5	40	0.74	66.0	1.000	0.3	0.3	0.1	1.0	0.741	1.350	596.4
6	0	12.92	67.0	1.203	0.3	0.3	0.1	1.0	1.094	1.323	470
6	10	7.41	66.0	1.209	0.3	0.3	0.1	1.0	1.029	1.421	496.3
6	20	2.56	65.0	1.228	0.3	0.3	0.1	1.0	0.982	1.535	525.7
6	30	1.68	65.0	1.033	0.3	0.3	0.1	1.0	0.795	1.343	558.9
6	40	1.02	65.0	0.901	0.3	0.3	0.1	1.0	0.667	1.216	596.4

S5: Flow performance metrics predictions

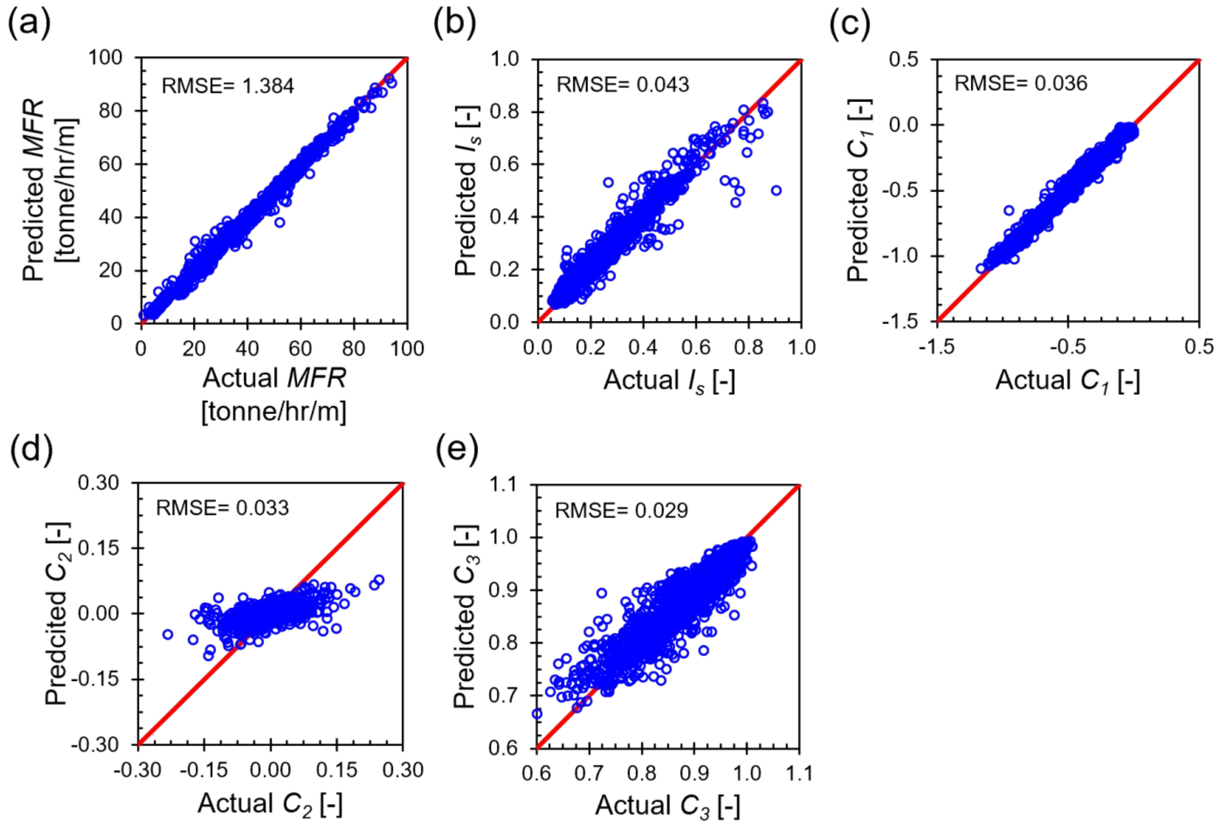

Figure S11. Flow performance metrics predictions for the training set: (a) MFR, (b) I_s, (c) C_1, (d) C_2, and (e) C_3.

S6: Guide for using the machine learning model in designing cross-scale attributes

This section details a step-by-step procedure of utilizing the machine learning design model to determine the cross-scale attributes required to achieve satisfactory flow performance metrics. This section features two illustrative examples under different scenarios: the first restricts adjustments to hopper operating conditions, while allowing flexibility in modifying particle-scale attributes. The second example allows freedom in adjusting hopper operating conditions, while particle-scale attributes are fixed. These examples were carried out using a straightforward application developed with very simple Python or Matlab scripts alongside the trained machine learning model. It prompts users to input their required average mass flow rate (MFR), including an allowance percentage (e.g., $\pm$ 5% of MFR), the maximum permissible smoothness index (I_s), and any additional requirements regarding flow pattern quadratic constants (C_1, C_2, C_3). In the main manuscript, we demonstrated that mass flow, funnel flow, and intermediate flow patterns can all result in intermittent unstable flows. Therefore, we focus herein on MFR and I_s to determine the combination of input variables that can achieve the desired flow performance. For this, the user inputs the magnitude of fixed, non-adjustable input variables, as well as the expected minimum and maximum values of flexible input variables, specifying the number of divisions within this range. The application then generates an array of all possible combinations of the input variables using Cartesian product. These combinations are fed into the trained machine learning model to predict flow performance metrics. Ultimately, the application presents these predictions through illustrative plots and identifies input combinations that meet the specified flow performance metrics.

I. Example 1:

This case limits adjustments to the hopper operating conditions, while the particle-scale attributes are adjustable. The design case requires an average mass flow rate MFR of 35 tonne/hr/m (note the unit m here indicates a unit length in hopper outlet length), an allowance percentage of $\pm$ 5% for MFR, and a maximum permissible smoothness index I_s of 0.25 for stable, smooth flow. The user is asked to enter all the information related to the cross-scale input variables as summarized in Table S11. The relative density D_r is chosen to have a fixed categorical variable of 1 (i.e., loose), indicating no prior compaction or densification of woody biomass. Accordingly, the application creates 65 possible combinations (i.e., $5 \times 13 \times 1 \times 1 \times 1 \times 1$), which are subsequently fed into the trained machine learning model for prediction.

Table S11. Cross-scale input variables information for Example 1.

Parameter	Adjustability	Minimum	Maximum	# of divisions
d_{50} [mm]	Flexible	2	6	5
MC [%]	Flexible	0	60	13
D_r [-]	Fixed	1 (loose)	1 (loose)	1
μ_w [-]	Fixed	0.268	0.268	1
θ [°]	Fixed	30	30	1
W [mm]	Fixed	75	75	1

Figure S12 shows the predicted MFR and I_s for the 65 combination of input variables. Note that the solid red lines in Figure S12 indicate the required MFR and I_s by the user, while the red shaded area in Figure S12a represents the $\pm 5\%$ allowance range for MFR. Based on these predictions, the application identifies and summarizes the combinations of input variables that meet the user's specifications, as shown in Table S12. These combinations serve as potential cross-scale attribute variables that can be utilized to achieve satisfactory flow performance.

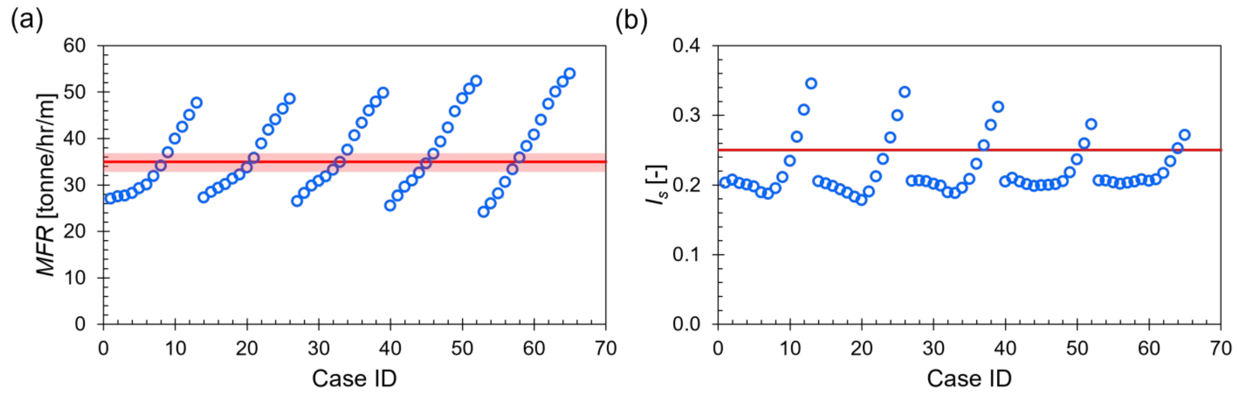

Figure S12. (a) Predicted average mass flow rate MFR and (b) predicted smoothness index I_s for the 65 combinations of input variables in Example 1.

Table S12. Summary of input variables combinations satisfying the flow performance metrics for Example 1.

Case ID	d50 [mm]	MC [%]	D_r [-]	μ_w [-]	θ [°]	W [mm]	MFR [tonne/hr/m]	I_s [-]	C_1 [-]	C_2 [-]	C_3 [-]
8	2	35	1 (loose)	0.268	30	75	34.14	0.195	-0.333	0.003	0.941
20	3	30	1 (loose)	0.268	30	75	33.70	0.178	-0.358	-0.003	0.944
21	3	35	1 (loose)	0.268	30	75	35.79	0.190	-0.357	-0.001	0.935
32	4	25	1 (loose)	0.268	30	75	33.27	0.189	-0.364	0.007	0.948
33	4	30	1 (loose)	0.268	30	75	34.91	0.188	-0.359	0.006	0.946
45	5	25	1 (loose)	0.268	30	75	34.55	0.199	-0.353	0.015	0.949
46	5	30	1 (loose)	0.268	30	75	36.64	0.200	-0.362	0.016	0.948
57	6	20	1 (loose)	0.268	30	75	33.35	0.203	-0.348	0.029	0.953
58	6	25	1 (loose)	0.268	30	75	35.89	0.204	-0.346	0.028	0.954

II. Example 2:

This case offers flexibility in modifying hopper operating conditions and limits adjustments to the particle-scale attributes. The design case requires an average mass flow rate

MFR of 25 tonne/hr/m, an allowance percentage of $\pm$ 5% for MFR, and a maximum permissible smoothness index I_s of 0.25 for stable, smooth flow. The user is asked to enter all the information related to the cross-scale input variables as summarized in Table S13. The relative density D_r is chosen to have a fixed categorical variable of 1 (i.e., loose), indicating no prior compaction or densification of woody biomass. Accordingly, the application creates 165 possible combinations (i.e., $1 \times 1 \times 1 \times 3 \times 5 \times 11$), which are subsequently fed into the trained machine learning model for prediction.

Table S13. Cross-scale input variables information for Example 2.

Fixed	Adjustability	Minimum	Maximum	# of divisions
d_{50} [mm]	Fixed	4	4	1
MC [%]	Fixed	10	10	1
D_r [-]	Fixed	1 (loose)	1 (loose)	1
μ_w [-]	Flexible	0.087	0.577	3
θ [°]	Flexible	20	60	5
W [mm]	Flexible	50	100	11

Figure S13 shows the predicted MFR and I_s for the 165 combination of input variables. Note that the solid red lines in Figure S13 indicate the required MFR and I_s by the user, while the red shaded area in Figure S13a represents the $\pm$ 5% allowance range for MFR. Based on these predictions, the application identifies and summarizes the combinations of input variables that meet the user's specifications, as shown in Table S14. These combinations serve as potential cross-scale attribute variables that can be utilized to achieve satisfactory flow performance.

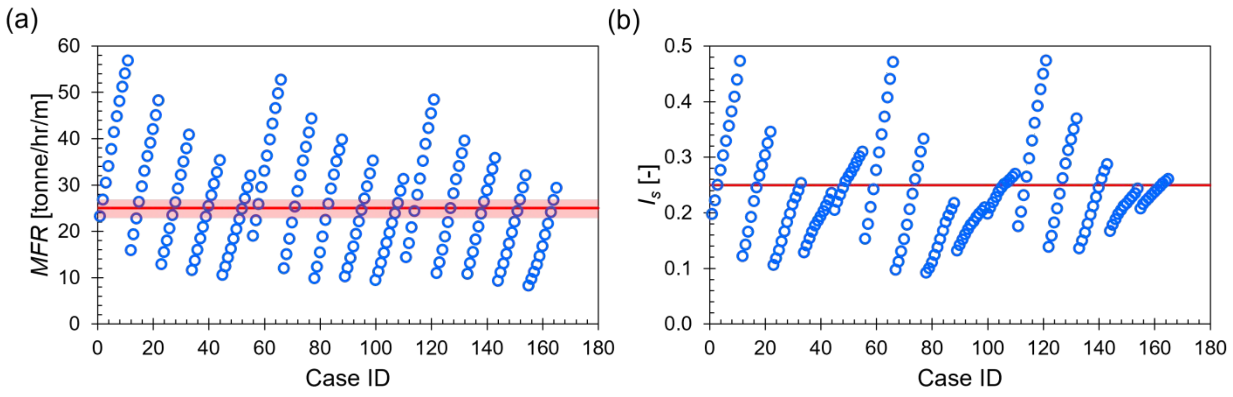

Figure S13. (a) Predicted average mass flow rate MFR and (b) predicted smoothness index I_s for the 165 combinations of input variables in Example 2.

Table S14. Summary of input variables combinations satisfying the flow performance metrics for Example 2.

Case ID	d50 [mm]	MC [%]	D_r [-]	μ_w [-]	θ [°]	W [mm]	MFR [tonne/hr/m]	I_s [-]	C_1 [-]	C_2 [-]	C_3 [-]
28	4	10	1 (loose)	0.087	40	75	26.249	0.183	-0.305	-0.001	0.910
40	4	10	1 (loose)	0.087	50	80	25.390	0.193	-0.327	-0.010	0.793
58	4	10	1 (loose)	0.332	20	60	25.795	0.210	-0.221	0.016	1.005
71	4	10	1 (loose)	0.332	30	70	25.269	0.178	-0.407	0.005	0.953
83	4	10	1 (loose)	0.332	40	75	25.865	0.152	-0.528	0.010	0.852
95	4	10	1 (loose)	0.332	50	80	24.548	0.184	-0.575	-0.002	0.754
151	4	10	1 (loose)	0.577	50	85	24.343	0.223	-0.975	-0.001	0.815
